# Post-trial access practice in malaria, tuberculosis, and NTDs clinical trial studies in Sub-Saharan African countries, quantitative study

**DOI:** 10.12688/openreseurope.18175.1

**Published:** 2024-10-07

**Authors:** Yemisrach Seralegne, Cynthia Khamala Wangamati, Rosemarie de la Cruz Bernabe, Ibrahim Mdala, Martha Zewdie, Hawult Taye Adane

**Affiliations:** 1 Clinical trial unit, Armauer Hansen Research Institute, ADDIS ABABA, Addis Ababa, 1005, Ethiopia; 2 Centre for Medical Ethics, Institute of Health and Society, Faculty of Medicine, University of Oslo, Norway, Oslo, 0450, Norway; 3 Department of General Practice,, Institute of Health and Society, University of Oslo, Oslo, Norway, Oslo, 0450, Norway

**Keywords:** post-trial access, clinical trial, TB, Malaria, NTDs

## Abstract

**Background:**

According to CIOMS, 2016 post-trial access (PTA) refers to the ethical imperative that requires the sponsor, researchers, and relevant public health authority, "to make available as soon as possible any intervention or product developed, and knowledge generated, for the population or community in which the research is carried out."

PTA is stipulated and recommended by different international research guidelines like CIOMS, and it was acknowledged that PTA should be accessible to those who actively participated in the trial study and the community and/or host country. Law, policy, and practical guidance for PTA has so far been vague but has recently attracted and increased attention in the context of benefit sharing of scientific research results with low- and middle-income countries (LMICs).

Even though the number of clinical trials conducted in the Sub-Saharan countries has increased in the past two decades, PTA plan and practice is underreported or very low.

**Objective:**

to evaluate PTA plan and implementation practice on TB, Malaria and NTD clinical trial studies conducted in the sub-Saharan African countries.

**Method:**

a quantitative, cross sectional study survey approach is used to evaluate the PTA plan and practice of PI, trial coordinators, and sponsors in Sub-Saharan African countries.

**Finding:**

misunderstanding of the term PTA, lack of plan, discussion, and arrangement on PTA between research stakeholders.

**Conclusion:**

PTA training should be prepared and facilitated for researchers, IRB members, PIs, funders, and sponsors; discussion and arrangement on PTA should be done before the conduct of the trial; and there should be written agreement between the parties to guarantee PTA to study participants and community after the end of the trial study.

## Introduction

### Background

Clinical trials are research studies performed in people aimed at evaluating a medical, surgical, or behavioral intervention. They are the primary way that researchers find out if a new treatment, like a new drug or diet or medical device (for example, a pacemaker) is safe and effective in people ( Vulcano, 2017). Often a clinical trial is used to learn if a new treatment is more effective and/or has less harmful side effects than the standard treatment ( NIH, 2023).

Most of the time clinical trials are conducted to test the safety and efficacy of new drugs, vaccines, medical devices, to test shorter duration of treatment, test a new dosage regimen, minimize side effects, optimize efficacy and safety, before or after marketing authorization ( Fekadu *et al.*, 2014).

 Clinical trials in Ethiopia and other African nations can considered to be in their embryonic stages. The share of studies registered from Africa is (in Clinicaltrials.gov) updated as of June 2017 is only 0.025%, although the region represents about 15% of the population of the world ( Fekadu *et al.*, 2014).

Within Africa, most of the clinical trials are conducted in South Africa (47.3%), which makes one of the largest financial investments on health and has a relatively well-developed health system. Only a limited number of clinical trials are registered from Ethiopia in trial registration sites, representing only 1.5% of all the studies from Africa ( Pratt *et al.*, 2012).

Post-trial access (PTA) refers to the ethical imperative that requires the sponsor, researchers, and relevant public health authority, "to make available as soon as possible any intervention or product developed, and knowledge generated, for the population or community in which the research is carried out ( *Clinical tiral in marketing authorization*, 2013) not to mention to research participants who still need the intervention ( *Declaration of Helsinki*, 2013).

 The PTA should be planned and documented in the study protocol. The execution plan should be reviewed and updated during the course of the clinical trial. PTA has various aspects. First, it refers to the provision of both knowledge and intervention, if any. Second, it refers to the potential beneficiaries, which could be research participants and/or the intended patient group in the community and/or country. Third, PTA provision to research participants must be free of charge, while PTA for the community and/or host country usually comes in the form of availability and accessibility of the medicinal product within a reasonable time.

Access to an investigational intervention is justified by the principle of beneficence, “which requires researchers and sponsors to safeguard the health of participants when it is in their power to do so.” It is also supported by the principle of distributive justice: participants, and by extension the community or host country, assist researchers in generating valuable data and, in return, researchers should ensure that participants receive needed care to safeguard their health.

Even though the number of clinical trials in LMICs has increased dramatically ( Van Roessel *et al.*, 2019), with most of the trials being funded, sponsored, or otherwise influenced by private or public entities from high-income countries, the lack of PTA introduces the risk of exploiting study participants in low and middle-income countries who otherwise have limited access to healthcare.

In this case, national ethics committees and the relevant drug regulation agencies must ensure that PTA is in place. While there is limited practical experience on PTA in clinical trials in LMICs, the concept of benefit-sharing, which acts as the theoretical foundation of PTA, is firmly stipulated in different international ethical guidelines.

The UNESCO Declaration on Bioethics and Human Rights, for example, highlights the duty of benefit-sharing pertaining to scientific and technological innovations, in particular with developing countries ( Chirac & Torreele, 2006; *UNESCO*, 2009);

The Declaration of Helsinki requires the sharing of benefits in medical research with the vulnerable group ( *Declaration of Helsinki*, 2013); and the CIOMS International Ethical Guidelines requires multi-stakeholder cooperation to make the studied intervention and the knowledge gained available to the population or community ( *CIOMS*, 2016).

Hence, ethical guidelines are clear that PTA, in the form of concrete plans and procedures for benefit-sharing and access (may it be in the form of reasonable pricing of an approved indication in the host country, free medicinal product for research participants, the provision of knowledge to the relevant patient population and the community), must be intrinsic to clinical trial planning. Despite these ethical guidelines, the general status quo of PTA is not encouraging: the provision of PTA is “rather the exception than the rule” ( Jimenez *et al.*, 2019; Schippe, 2015).

In line with the above principles, this manuscript will explore PTA planning and implementation on TB, Malaria, and NTD clinical trials in Sub-Saharan African countries.

## Methods

### Study design

The study is a descriptive cross-sectional study. Cross-sectional surveys were used to explore PTA planning and implementation on TB, Malaria, and NTD clinical trials in Sub-Saharan African countries.

### Study setting

The study was conducted by sending emails with a link to the online survey questionnaires to respondents who either conduct/have conducted clinical trials in sub-Saharan countries.

### Sample and sampling strategy

The study respondents were principal investigators (PI), Co-PI, sponsors, researchers, and clinical trial coordinators conducting clinical trial research in TB, Malaria, and NTDs in sub-Saharan African countries. Purposive sampling was used to select the study sample. The researchers collected 300 study respondents’ contact information from two clinical trial study registry sites namely called clinical trials.gov and Pan African clinical Trial registry (PACTR). The study questionnaire was sent to all the 300 study respondents, only 37 respondents completed the questionnaire.

### Data collection tool and procedures

A close ended survey questionnaire was developed based on the objective of the study; to know their role in clinical trials, to explore PTA plans, and respondents’ need for PTA training. The survey questionnaire was pre-tested amongst AHRI researchers for its clarity and distributed to the study participants through their email. Initially, a longer questionnaire with 17 questions on socio-demographics, PTA knowledge, PTA plans, discussions and arrangements, PTA implementation, stakeholder involvement, trial products, and roles was sent out. Unfortunately, the response rate was low and as such we shortened the questionnaire to encourage study participation. Responses were saved on the AHRI server.

### Data management and analysis

(“StataSE 18”) was used to perform descriptive statistics in the form of frequencies and percentages, on anonymous data to maintain participants confidentiality (" Stata 18 software,"). The Fisher’s exact test used to assess the associations between categorical variables. The findings are presented in tables and graphically. We considered p-values less than 0.05 as statistically significant. Though we used STATA version 18 software, we shared the dataset with Excel sheet- which can be used to perform similar descriptive analysis, or it can be exported to other freely available statistical software’s like R. The R- or R Studio software is freely alternative available software that can be downloaded to most operating systems including Microsoft Windows, Linux, Mac OS, used to replicate the study result (" R Core Team (2021)R:," ; " R Studio Team (2020),").

### Ethical considerations

This study received ethical approval from the Armauer Hansen Research Institute (AHRI) and the All-African Tuberculosis, Leprosy Treatment, Rehabilitation, and Training Centre (AHRI/ALERT IRB) with approval reference number PO/43/20 dated on 21/01/2021. Additionally, the Ministry of Education (MOE) National Ethical Review Board (NERB) of Ethiopia granted approval with reference number MOSHE/RD/04/246/84/21 dated on 10/06/2021. The study also got renewal for approval from the Ministry of Education (MOE) National Ethical Review Committee of Ethiopia with the reference number of MOSHE/RD/03/246/505/22 dated on 08/06/2022. Written informed consent was obtained from all study participants prior to their participation in the study. Within the data collection procedures, the applicant strictly maintained the participant’s confidentiality by using the study code number as identification for each participant for anonymity.

## Results

### Characteristics of the study population

Three hundred potential study participants were invited to participate in the study survey questionnaires. As illustrated in “ Table 1”, Thirty-seven study participants gave a complete response to the survey questionnaires given. Out of the 37% study participants, principal investigators were 21 (56.8%), sponsors were 4 (10.8%), study coordinators were 12 (32.4%), with majority of participants being based in sub-Saharan Africa (56.8%).

**Table 1.** Distribution of the study participants stratified by their roles (N = 37).

	Role of study participants			Total	*P*-value
	Principal investigator	Sponsor	Study co-ordinator		
**Lead institute**					0.01
Pharmacitical company/ Partners	3 (33.3)	4 (44.4)	2 (22.2)	9 (24.3)	
Public Health/ Research Institute	6 (85.7)	0 (0.0)	1 (14.3)	7 (18.9)	
University	12 (57.1)	0 (0.0)	9 (42.9)	21 (56.8)	
**Funder**					0.07
LSHTM	2 (28.6)	0 (0.0)	5 (71.4)	7 (18.9)	
Others	19 (63.3)	4 (13.3)	7 (23.3)	30 (81.1)	
**Origin**					0.02
Africa	16 (76.2)	0 (0.0)	5 (23.8)	21 (56.8)	
Collaborative	2 (33.3)	2 (33.3)	2 (33.3)	6 (16.2)	
Not based in Africa	3 (30.0)	2 (20.0)	5 (50.0)	10 (27.0)	
**Site**					0.13
Multi-center	5 (45.5)	3 (27.3)	3 (27.3)	11 (29.7)	
Single-Center	16 (61.5)	1 (3.9)	9 (34.6)	26 (70.3)	
**PTA arrangement**					0.72
Yes	12 (57.1)	3 (14.3)	6 (28.6)	21 (56.8)	
No	9 (56.3)	1 (6.3)	6 (37.5)	16 (43.2)	
**PTA training**					0.46
Yes	13 (50.0)	3 (11.5)	10 (38.5)	26 (70.3)	
No	8 (72.7)	1 (9.1)	2 (18.2)	11 (29.7)	

**Figure 1.**
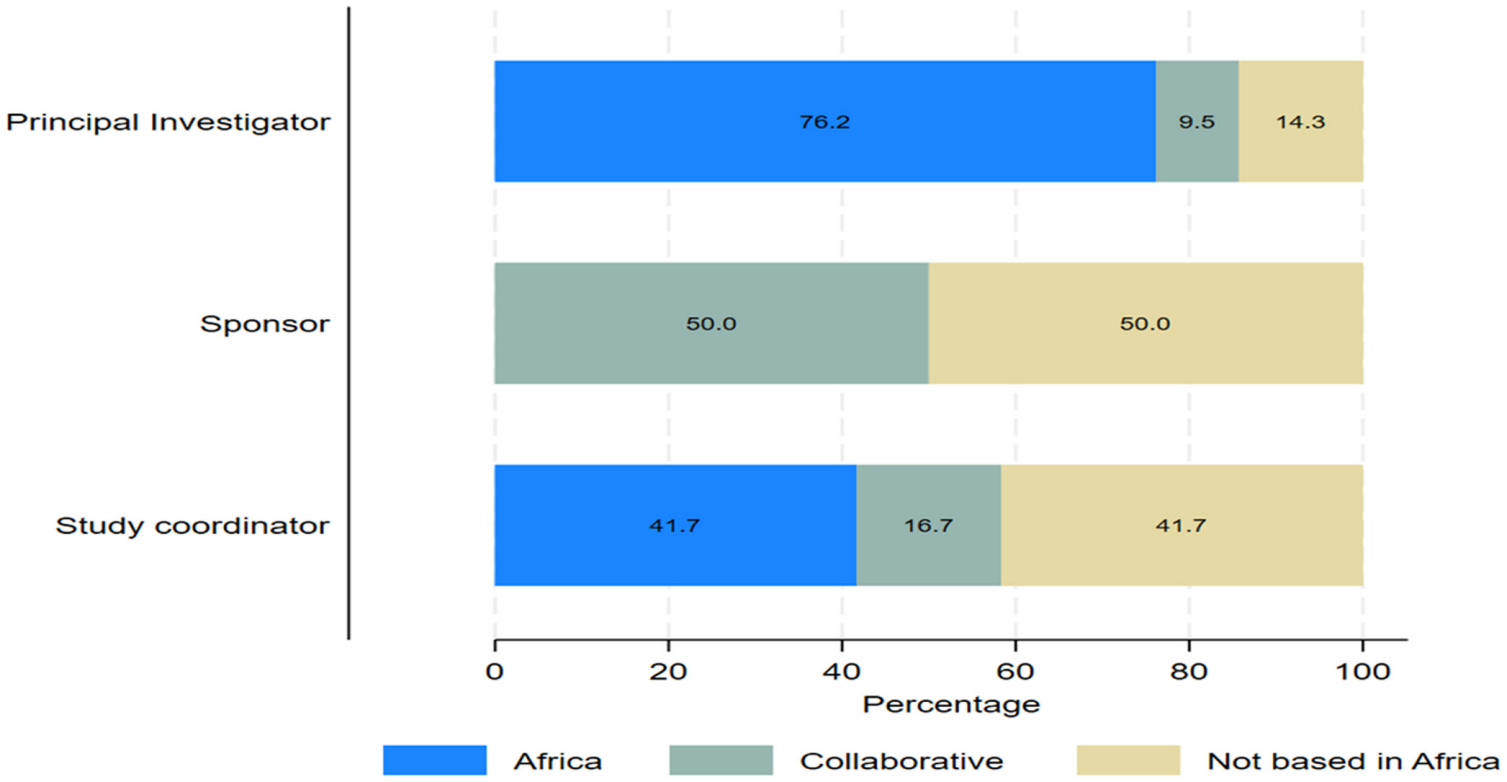
Stacked bar graph showing the distribution of key roles by origin.

Majority of the respondents were from sub-Saharan Africa as indicated by “ Figure 1”. Most of the principal investigators (76.2%) were from Africa. Sponsors were equally distributed between collaboratives and not based in Africa. Africa and not based in Africa contributed 41.7% of the study coordinators each with the rest being collaborative study coordinators.

**Figure 2.**
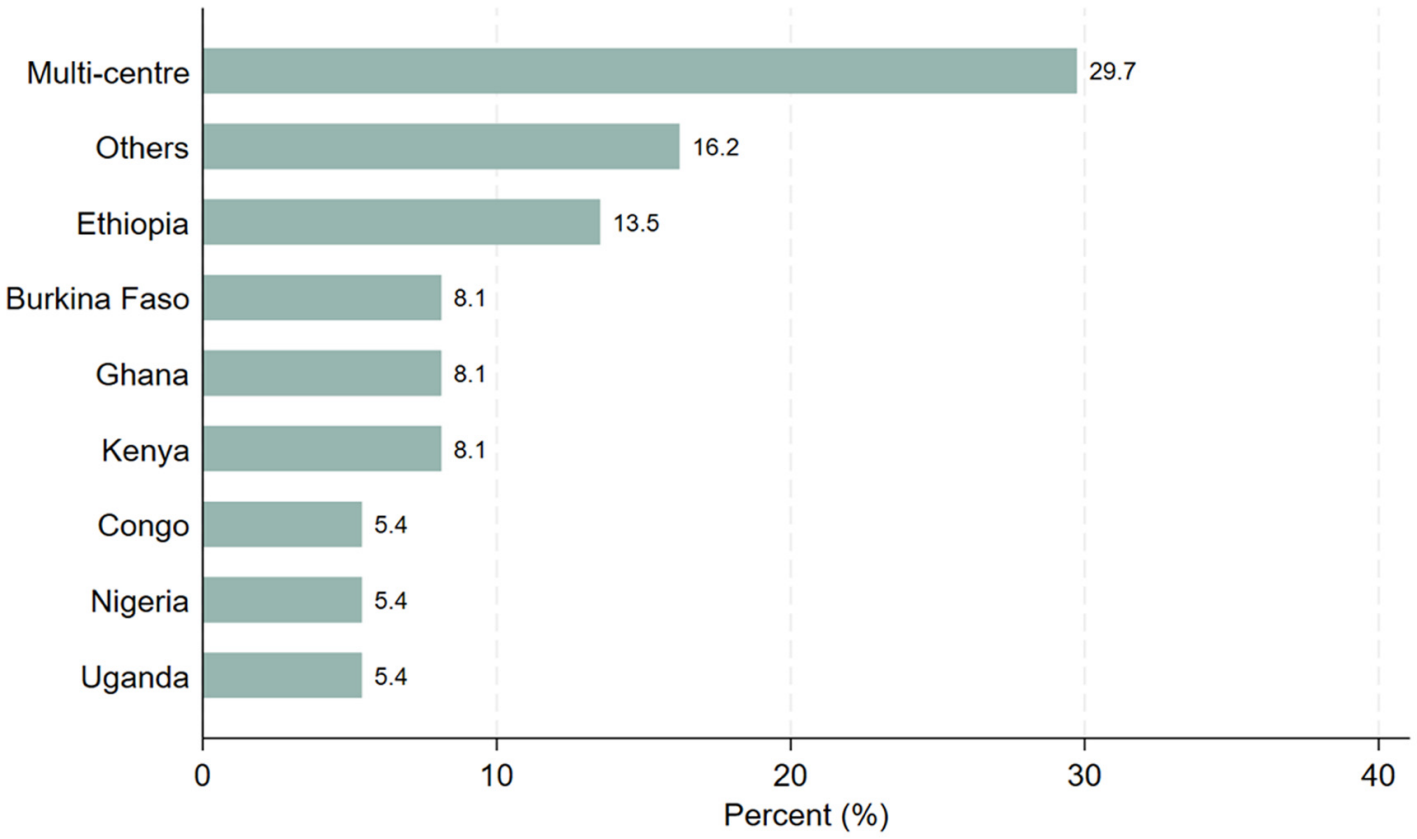
Distribution of the study participants by site (N=37).

From those, 70% of them conducted single centered clinical trial while 30% of the respondents conducted multi-center clinical trials in the sub-Saharan African countries as indicated by Figure 2.

**Figure 3.**
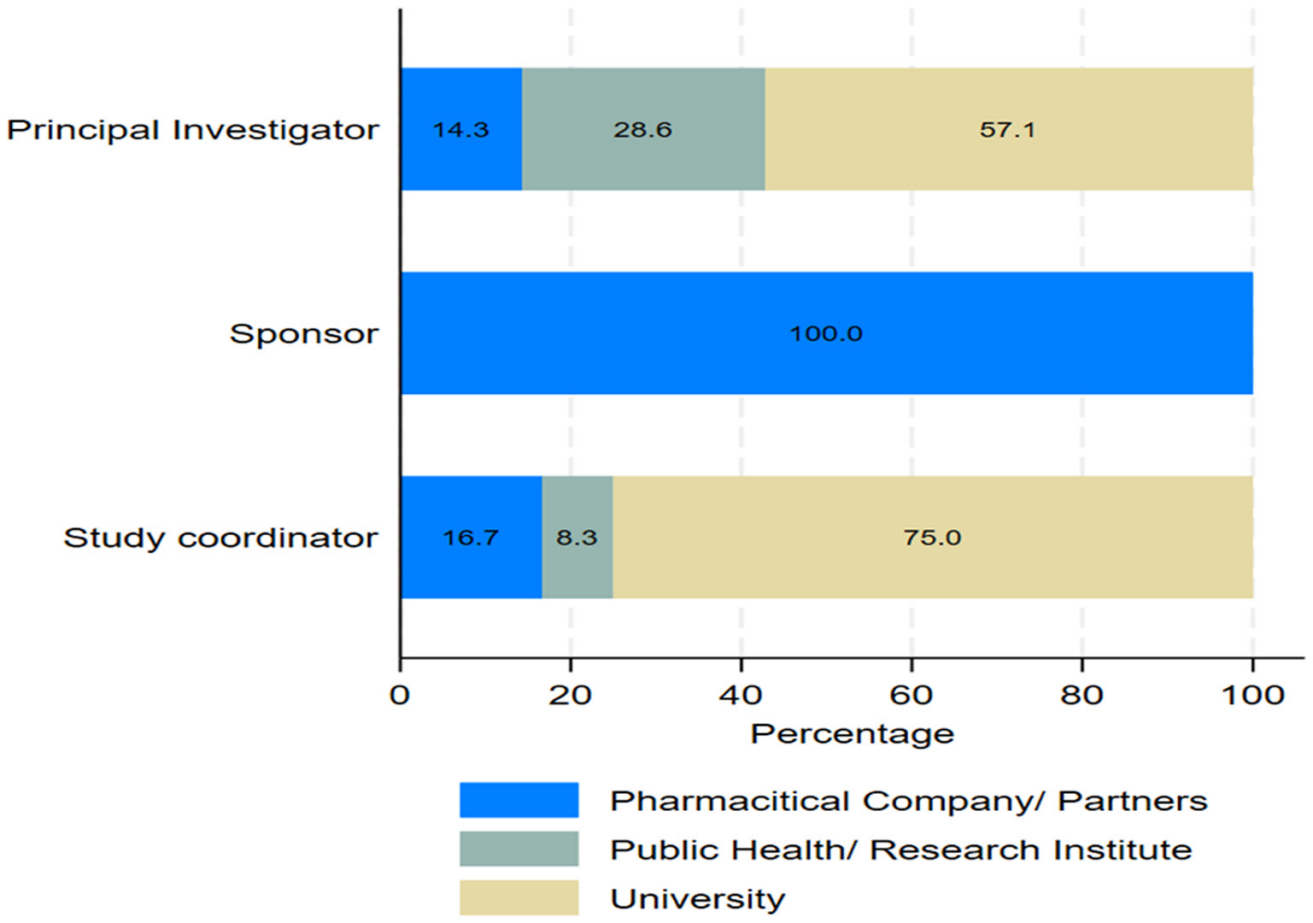
Stacked bar graph showing the distribution of key roles by lead institutions.

As shown by Figure 3, 57% and 75% of the principal investigators and study coordinators were from universities respectively, whereas all sponsors were from pharmaceutical companies.

### Post-trial access plans and arrangements

**Figure 4.**
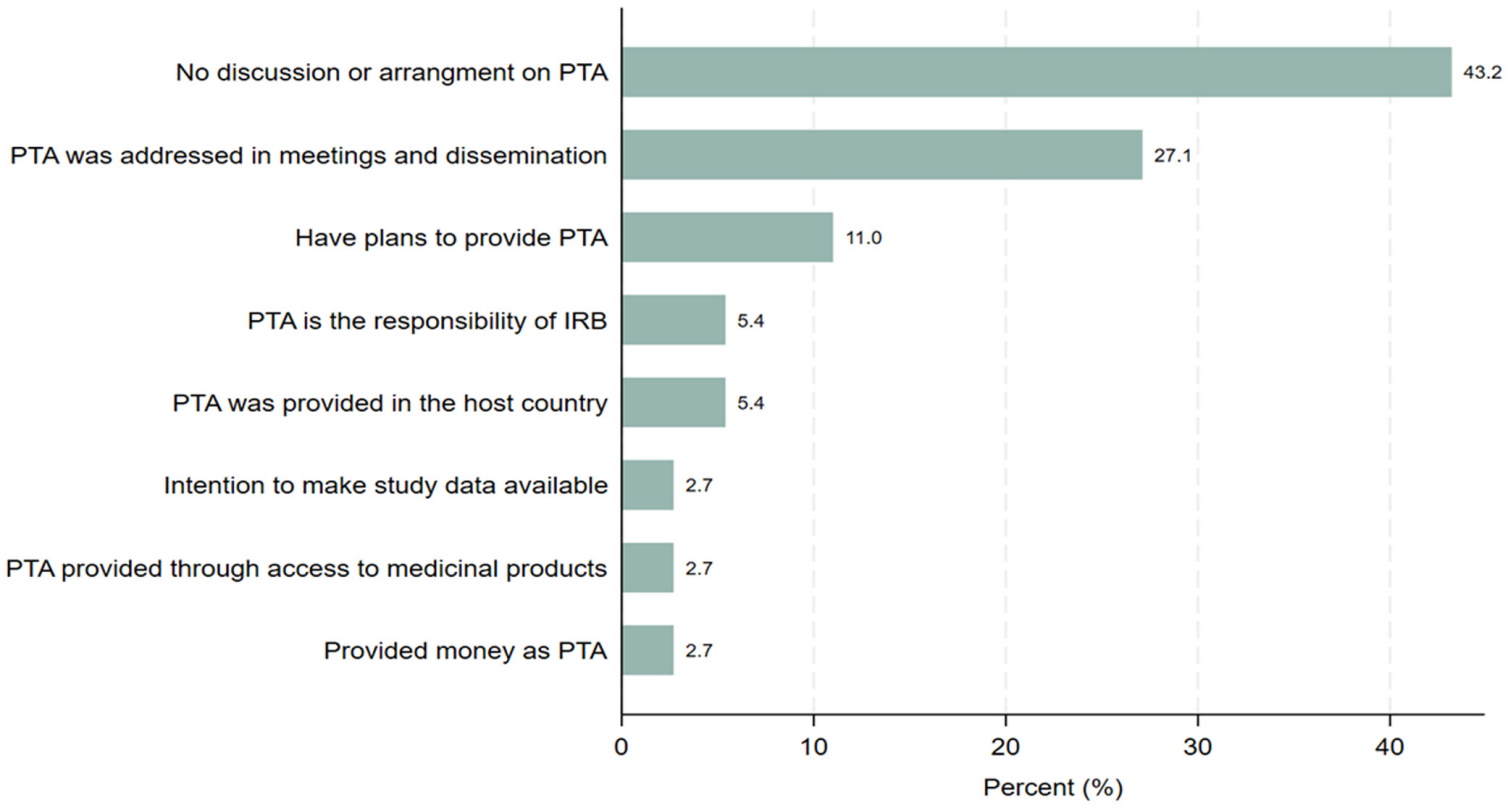
Distribution of the responses of PTA plans, discussion or arrangements made.

Respondents were asked whether they provided PTA plans, discussions or arrangements were made in their clinical trials and if yes, in what form. A total of 21 (56.8 %) study participants stated that they provided PTA plans, discussions, or arraignments. Of the 37 study participants, 21 (56.8%) provided varying responses on PTA plans, discussions, and arrangements ( Figure 4). Ten study participants representing 27.1% of the sample population said that PTA was addressed in the form of meetings and dissemination of study findings, 4 (11%) had plans to provide PTA in the form of preferential price or freely, 2 (5.4%) replied that provision of PTA is task of the IRB or the regulatory authorities while a further 2 (5.4 %) said that PTA was not provided because the product (medication for acute malaria) was available in the host country. Our findings also showed that 1 respondent (2.7%) said that they will make study data available, 1 (2.7%) replied that PTA was provided in the form of access to the medicinal product by the community and another respondent said that they would provide money as PTA. However, 16 (43.2%) of the participants responded that there was no discussion and arrangements made on PTA related to trial studies conducted on malaria, Tuberculosis or NTDs in Sub-Saharan African countries. The findings demonstrate that planning and provision of PTA is neglected even though the number of clinical trial studies conducted in the sub-Saharan Africa countries has been steadily increasing with in the last two decades.

### PTA training needs

**Figure 5.**
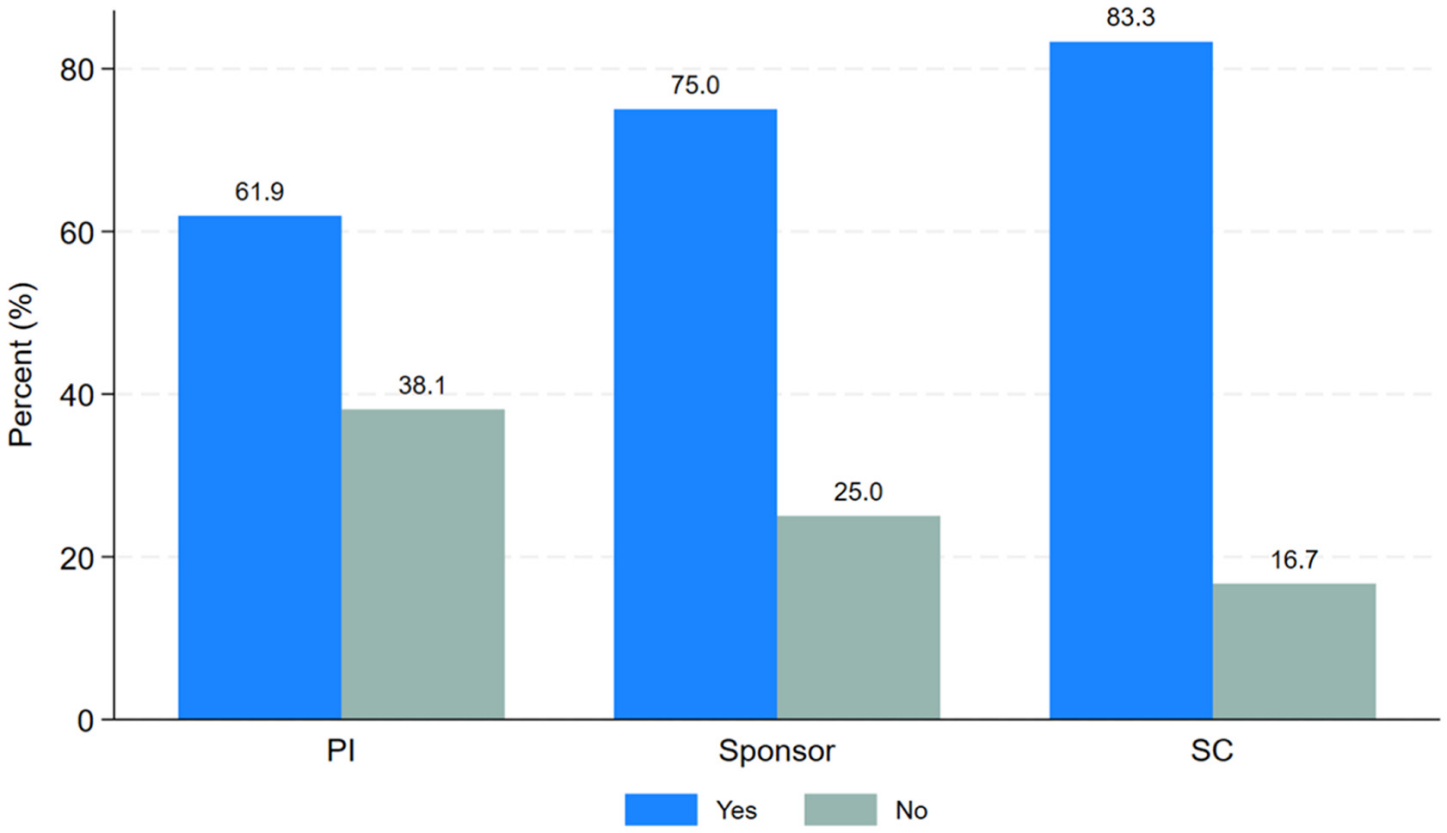
Clustered bar graph showing the distribution of key roles of the study participants by PTA.

Study participants were asked if they required PTA training. Twenty-six respondents (70.3%) are willing to attend training on PTA arrangements for future clinical trial studies. However, 6 respondents (16.2%) were not willing to attend PTA training while 5 respondents (13.5%) did not declare their interest for the PTA training. Thus, most of the respondents are willing to learn about PTA arrangements and implementation. As indicated by “ Figure 5”, Most of the PIs (61.9%), sponsors (75%) and study coordinators (83.3%) stated that they would like to receive PTA training.

## Discussion

The findings of this study are interesting since though the majority of the participants declared consideration of PTA, only 10% said that dissemination of knowledge is considered, only 11% said that differential pricing is considered (i.e., PTA as community availability and accessibility), and only 2.7% said that PTA will be provided in the form of access of the medicinal product to the community (what this meant is not exactly clear, i.e., whether the medicinal product will be made accessible freely or if the meaning is the same as the previous group of making the medicinal product accessible via differential pricing). These are bleak numbers but is very much reflective of earlier findings such as that of Bernabe, *et al.* where PTA was declared by the majority of clinical trial PIs, though a closer look at their responses showed that there was no PTA in the form of access to the medicinal product by either the research participants or the community and/or host country ( Jimenez *et al.*, 2019).

The PTA plan should be requested by IRB members during the review process of trial documents. The fact that PTA remains an exception than the rule among the participants point to the direction that this is not the case. That there is no pan-African (or international) platform to follow up on whether researchers implement their PTA plans once the research is completed makes oversight difficult.

In the case of at least some if not most of the Sub-Saharan countries, the absence of regulation and follow-up on the implementation process of PTA presents a significant challenge to its feasibility. The challenges related to PTA stem from a lack of knowledge, mutual agreement, and commitment from those responsible for ensuring the rights, safety, and well-being of study participants and the community.

Addressing these challenges can be achieved through PTA training for researchers, sponsors, and IRB members to update their perspectives on PTA and to consider it in the protocol development and review process. Active participation by research stakeholders in the planning, process, and implementation of PTA can maximize its feasibility in the region and help ensure the rights and benefits of research participants and their communities/countries.

## Limitations

First, the low response rate from selected study participants and the trials they represented hindered our ability to evaluate whether the studies considered had plans for the provision and implementation of PTA. As a result, we could not conclude whether the selected trial studies had a PTA plan and implementation strategy in place before the trial was conducted.

Second, the incomplete response rate on the survey questionnaires was one of the main challenges we faced in analyzing and evaluating the relationship between the planning and implementation of PTA practices in selected clinical trial studies in Sub-Saharan African countries.

## Conclusion and recommendations

Training on PTA should be provided to research stakeholders to fill the knowledge gap. The research guidelines of each country should be amended to include a PTA plan and ensure access to study participants and communities who need the medicinal product. Additionally, distributive justice and beneficence require that there should be a mutual agreement between the researcher, funder, sponsor, and host country to provide PTA to the study participants and communities.

## Data Availability

The data for this article consists of bibliographic references, which are included in the References section. Zenodo: Post-trial access practice in Malaria, Tuberculosis, and NTDs Clinical Trial studies in Sub-Saharan African countries, quantitative study. https://zenodo.org/doi/10.5281/zenodo.13752053 Figure 1. Stacked bar graph showing the distribution of key roles by origin. Figure 2 Distribution of the study participants by site. Figure 3. Stacked bar graph showing the distribution of key roles by lead institutions. Figure 4. Distribution of the responses of PTA plans, discussion or arrangements made. Figure 5. Clustered bar graph showing the distribution of key roles of the study participants by PTA training. Table 1. Distribution of study participants stratified by their role. Information sheet for study participants. PTA data Excel format. Raw data survey 1 Raw data survey 2 Study survey questionnaires Data are available under the terms of the Creative Commons Attribution 4.0 International license (CC-BY 1.0).
