# Post-trial access practices in conducted clinical trials for Malaria, Tuberculosis, and Neglected Tropical Diseases (NTDs) across Sub-Saharan African countries:  A quantitative study

**DOI:** 10.12688/openreseurope.18175.4

**Published:** 2025-06-23

**Authors:** Yemisrach Seralegne, Cynthia Khamala Wangamati, Rosemarie de la Cruz Bernabe, Ibrahim Mdala, Martha Zewdie, Hawult Taye Adane

**Affiliations:** 1 Clinical trial unit, Armauer Hansen Research Institute, Addis Ababa, 1005, Ethiopia; 2 Centre for Medical Ethics, Institute of Health and Society, Faculty of Medicine, University of Oslo, Norway, Oslo, 0450, Norway; 3 Department of General Practice, Institute of Health and Society, University of Oslo, Oslo, Norway, Oslo, 0450, Norway

**Keywords:** post-trial access, clinical trial, TB, Malaria, NTDs

## Abstract

**Background:**

According to the Council of International Organizations and Medical Sciences (CIOMS) 2016, post-trial access (PTA) refers to the ethical imperative that requires the sponsor, researchers, and relevant public health authority, "to make available as soon as possible any intervention or product developed, and knowledge generated, for the population or community in which the research is carried out." Law, policy, and practical guidance for PTA has so far been vague but has recently attracted and increased attention in the context of benefit sharing of scientific research results with low- and middle-income countries (LMICs).

Although the number of clinical trials conducted in the Sub Saharan (SSA) countries has increased in the past two decades, plans and practices for PTA are underreported. The study examines PTA planning and implementation in clinical trials focused on Tuberculosis (TB), Malaria, and Neglected Tropical Diseases (NTDs) in Sub-Saharan African countries, conducted between 2008 and 2019.

**Objective:**

The study aims to identify gaps in PTA planning and implementation, highlight challenges, and suggest strategies for improving access to trial interventions and knowledge post-research.

**Method:**

A quantitative, cross-sectional study was conducted, using a self-administered online questionnaire to assess the PTA planning and implementation practices of Principal Investigators (PIs), co-PIs, trial coordinators, and sponsors involved in clinical trials in malaria, tuberculosis and NTDs across Sub-Saharan African countries. Of the 300 invited potential participants, 37 provided complete responses.

**Findings:**

A large proportion (43%) of the study respondents did not provide PTA plans for TB, Malaria, and NTDs in clinical trials. The findings highlight an overall lack of formalized PTA policies and commitments in clinical trials for TB, Malaria, and NTDs in Sub-Saharan Africa. Most of the study participants (70.3%) expressed the need for PTA training.

**Conclusion:**

Although the study offers valuable insights into PTA planning and practices, its generalizability may be limited by factors such as geographical and disease focus, reliance on self-reported data, and stakeholder representation. Despite these limitations, the study underscores an urgent need for structured PTA policy training programs, stakeholder collaboration, and effective training. Its findings can serve as a foundation for further research and policy development to enhance PTA in LMICs.

## Introduction

### Background

Clinical trials are research studies performed in people aimed at evaluating a medical, surgical, or behavioral intervention. They are the primary way that researchers find out if a new treatment, like a new drug or diet or medical device (for example, a pacemaker) is safe and effective in people ( Vulcano, 2017). A clinical trial is often used to learn if a new treatment is more effective and/or has less harmful side effects than the standard treatment ( NIH, 2023).

Africa is home to 17% of the world’s population and carries 25% of the global disease burden ( Hwenda *et al.*, 2022). However, it contributes less than 3% of worldwide clinical trials conducted on the continent ( Hwenda *et al.*, 2022). Studies have shown that this is mainly due to limited clinical trial capacity, which is attributed to inadequate funding, poor research infrastructure, and a lack of trained personnel, including clinical trialists and laboratory researchers ( Vischer, 2016). Furthermore, regulatory challenges, including ethical concerns, significantly hinder the implementation and expansion of clinical trials in Africa ( Ndembi *et al.*, 2024). Consequently, PTA practices are not as well established or widespread in Africa compared to other continents. Enhancing clinical trial application and review capabilities in African countries is crucial for revitalizing the continent's role in managing communicable and non-communicable diseases. It significantly improves PTA plans and practices among stakeholders and enhances the region’s preparedness for frequent pandemic onsets ( Ogunleye *et al.*, 2020).

Declaration of Helsinki, article 34 PTA states, “In advance of a clinical trial, sponsors, researchers, and host country governments should make provisions for post-trial access for all participants who still need an intervention identified as beneficial in the trial. This information must also be disclosed to participants during the informed consent process.” ( DoH, 2013). The Declaration of Helsinki has been revised; there is a recent version of 2024 ( DoH, 2024). Therefore, PTA should be planned and documented in the study protocol. The execution plan should be reviewed and updated during the clinical trial. PTA has various aspects. First, it refers to providing knowledge and intervention, if any. Second, it refers to the potential beneficiaries, which could be research participants and/or the intended patient group in the community and/or country. Third, PTA provisions for research participants must be free of charge. PTA for the community and/or host country usually comes in the form of availability and accessibility of the medicinal product within a reasonable time. Access to an investigational intervention is justified by the principle of beneficence, “which requires researchers and sponsors to safeguard the health of participants when it is in their power to do so.” ( CIOMS, 2016) It is also supported by the principle of distributive justice: participants, and by extension, the community or host country, assist researchers in generating valuable data, and, in return, researchers should ensure that participants receive the needed care to safeguard their health.

Even though the number of clinical trials in LMICs has increased dramatically ( Van Roessel *et al.*, 2019), most of the trials are funded, sponsored, or otherwise influenced by private or public entities from high-income countries. The lack of PTA introduces the risk of exploiting study participants in low- and middle-income countries who otherwise have limited access to healthcare ( Singh *et al.*, 2019). In this case, national ethics committees and the relevant drug regulation agencies must ensure PTA is in place. While there is limited practical experience on PTA in clinical trials in LMICs, the concept of benefit-sharing, which acts as the theoretical foundation of PTA, is firmly stipulated in different international ethical guidelines ( Singh *et al.*, 2019). The UNESCO Declaration on Bioethics and Human Rights, for example, highlights the duty of benefit-sharing pertaining to scientific and technological innovations, in particular with developing countries ( Chirac & Torreele, 2006; UNESCO, 2009).

The Declaration of Helsinki requires the sharing of benefits in medical research with the vulnerable group ( DoH, 2013), and the CIOMS International Ethical Guidelines require multi-stakeholder cooperation to make the studied intervention and the knowledge gained available to the population or community ( CIOMS, 2016).

Hence, ethical guidelines are clear that PTA, in the form of concrete plans and procedures for benefit-sharing and access (may it be in the form of reasonable pricing of an approved indication in the host country, free medicinal product for research participants, the provision of knowledge to the relevant patient population and the community), must be intrinsic to clinical trial planning. Despite these ethical guidelines, the general status quo of PTA is not encouraging: the provision of PTA is “rather the exception than the rule” ( Jimenez *et al.*, 2019; Schippe, 2015).

In line with the above principles, this manuscript will explore PTA planning, type, and implementation on TB, Malaria, and NTDs clinical trials across Sub-Saharan African countries. For the purposes of this study, we define PTA arrangements or plans as preliminary discussions, commitments, and strategies developed before or during the trial to ensure post-trial access. It includes, but not limited to: (i)financial commitments for PTA, (ii) data and knowledge sharing plans, (iii) stakeholder and community engagement activities, including meetings with government agencies, regulators, or local organizations to discuss PTA expectations, (iv) informal and formal PTA discussions and commitments among sponsors, investigators, and health authorities, (v) addressing ethical review requirements related to PTA, (vi) collaboration between academic institutions and funding agencies to discuss PTA feasibility, (vii) negotiation of intellectual property (IP) rights or licensing agreements, where applicable, and (viii) inclusion of PTA considerations in trial protocols or informed consent documents.

We define PTA implementation as concrete actions after trial completion to ensure access to the tested interventions and/or knowledge. Examples include but are not limited to: (i) preferential pricing strategies for products in trial or host countries, (ii) conducting dissemination meetings with stakeholders, (iii) publishing results in open-access journals and popular literature to ensure knowledge sharing, (iv) securing regulatory approvals for the compassionate use of the intervention, (v) integration of the intervention into national health programs within a defined timeframe, (vi) technology transfer or capacity-building initiatives in the host country, (vii) distribution of educational materials, such as training guides or films, to relevant stakeholders, and (viii) post-implementation monitoring and/or evaluation to assess the uptake of impact of PTA efforts. 

## Methods

### Study design

The study is a descriptive cross-sectional study. A self-administered online questionnaire was used to explore PTA planning and implementation practices among principal investigators, sponsors, and study coordinators who implemented TB, Malaria, and NTDs clinical trial studies between 2008 and 2019. The searches are conducted from November 1, 2021, to January 31, 2022, using the Clinical trial.gov and Pan African clinical trial registry. The search terms include tuberculosis, malaria and neglected tropical diseases (NTDs), focusing on clinical trial studies conducted between 2008 to 2019 in Sub-Saharan African countries. The results are limited to trials registered in either the Clinical trial.gov or Pan African clinical trial registry. ( PACTR; tiral.gov).

We identified 242 completed clinical trial studies from the registers. From the identified studies, we selected a total of 300 participants and invited them to participate in the study. From the registry, 110 participants were registered as PI and sponsor, 122 were registered as PIs, and 78 were registered as trial coordinators. A total of 37 study participants agreed to participate by filling out the questionnaires. Of the 37 participants, 21 (56.8%) were principal investigators, 4 (10.8%) were sponsors, and 12 (32.4%) were study coordinators. Most participants were based in sub-Saharan African countries (56.8%).

We developed and distributed a survey questionnaire to collect information on the PTA, types, and implementation practices of PIs, Sponsors, and trial coordinators who conducted clinical trials on TB, Malaria, or NTDs across Sub-Saharan Africa based on a literature review of the literature ( Cook, 2014).

### Study setting

We sought clinical trial studies on Malaria, TB, and NTDs within SSA conducted between 2008 and 2019. We sought these studies in the clinicaltrials.gov and Pan African Clinical Trial Registry.

### Sample and sampling strategy

The respondents were principal investigators (PI), Co-PI, sponsors, researchers, and clinical trial coordinators who conducted clinical trial research in TB, Malaria, and NTDs between 2008 and 2019 within sub-Saharan African countries. Purposive sampling was used to select the study sample. The recruitment of study participants entailed sending emails to PIs, co-PIs, sponsors, researchers, and trial coordinators who were involved in clinical trials on Malaria, TB, & NTDs between 2008 and 2019 within Sub-Saharan African countries. These individuals were registered on the clinicaltrials.gov or Pan African Clinical Trial Registry websites. We retrieved their contact information from the registries and emailed consent forms and questionnaires. Some of the contact information was not useful as some participants had moved on to other organizations, making it difficult to trace them and, as such, limiting the response rate. Only 37 respondents completed the questionnaire. Online surveys are known for poor response rates in low- and middle-income countries due to internet connectivity issues ( Nayak & Narayan, 2019).

The selection of 300 participants was based on the number of completed clinical trial studies identified from two clinical trial registries, totaling 242 studies as shown in Table 1. From these studies, we compiled a list of Principal Investigators (PIs), trial coordinators, and sponsors who were eligible to provide insights into post-trial access (PTA) practices. The final sample size of 300 was determined based on the total number of unique individuals associated with these trials, ensuring a diverse and representative pool of participants across different roles in clinical trial implementation.

**Table 1.** Search strategy.

Name of the data base	Date the search conducted	Search words	Conducted year	How combined the search term	Number of records
Clinical trial.gov & Pan African clinical trial registry	November 1, 2021 – January 31, 2022	Tuberculosis	2008 – 2019	Completed clinical trial study in Sub-Sahara African countries	70
Clinical trial.gov & Pan African clinical trial registry	November 1, 2021 – January 31, 2022	Malaria	2008 – 2019	Completed clinical trial study in Sub-Sahara African countries	114
Clinical trial.gov & Pan African clinical trial registry	November 1, 2021 – January 31, 2022	Neglected tropical diseases (NTDs)	2008 – 2019	Completed clinical trial study in Sub-Sahara African countries	58

Despite our repeated efforts to engage study participants through more than two reminder emails and outreach via social media platforms such as Facebook, LinkedIn, and Twitter the response rate remained low, with only 37 out of 300 participants completing the self-administered questionnaire. This low response rate reflects common challenges associated with conducting online surveys in low- and middle-income countries (LMICs). The following factors may have contributed to the limited participation:

1. **Internet connectivity and accessibility issues:** Many researchers and stakeholders in Sub-Saharan Africa face intermittent internet access, which may have hindered their ability to receive, complete, and submit the online survey.
2. **Time constraints and competing priorities:** PIs, sponsors, and trial coordinators are often engaged in multiple research activities, limiting their availability to respond to surveys.
3. **Email deliverability and engagement challenges:** Some invited participants may not have actively checked or engaged with their email invitations, contributing to the low response rate. Additionally, participants may have changed their email addresses due to shifts in roles or responsibilities after the research period, or because of changes in their workplace, which often leads to a change in their work email address.

Despite the lower-than-expected response rate, the study provides valuable insights into PTA planning and implementation. The findings highlight critical gaps and reinforce the need for further exploration through mixed-method approaches, such as qualitative interviews or targeted stakeholder engagement, to enhance participation in future studies. In our study, we initially invited 300 eligible participants, of whom 37 provided complete responses to the survey. Therefore, for all response-based analyses, we used total number as the denominator when calculating percentages.

The search for TB, Malaria, and NTDs clinical trial studies was conducted from November 1, 2021, to January 31, 2022. The keywords used for the study were TB, Malaria, NTDs, trial ID, scientific trial title, disease name, clinical trial start time, completed time, recruitment country, PI contact address, sponsor email address, and 1st author name and address.

### Data collection tool and procedures

Based on the research objective, a questionnaire with 17 questions on socio-demographics, PTA knowledge, PTA plans, discussions and arrangements, PTA implementation, stakeholder involvement, trial products, and roles were sent out. The survey questionnaire was pre-tested amongst the Armauer Hansen Research Institute (AHRI) researchers to check if the developed tool is linguistically meaningful, if all necessary questions are asked in the right way, or if any other challenges may have been overseen or undetected during the process of tool development. Afterwards, the questionnaire was distributed to the study participants through their email addresses. Unfortunately, the response rate was low, so we shortened the questionnaire to encourage study participation. The participant responses were stored on the AHRI server.

The shortened questionnaire contained a few questions on (i) socio-demographics, (ii) roles in the conducted clinical trials, (iii) PTA plans, types, and implementation practices, & (iv) the need for PTA training. It was combined with information sheets and consent forms and sent to 300 email addresses.

### Data management and analysis

StataSE18 ( https://www.stata.com) software was used to perform descriptive statistics in the form of frequencies and percentages, on anonymous data to maintain participants confidentiality The Fisher’s exact test assessed the relationship between two categorical variables. The findings are presented in tables and graphically. We considered p-values less than 0.05 as statistically significant. Though we used STATA version 18 software, we shared the dataset with Excel sheet- which can be used to perform similar descriptive analysis, or it can be exported to other freely available statistical software’s like R ( RStudio Team, 2020). The R- or RStudio software is freely available language and environment for statistical computing and graphics that can be downloaded to most operating systems including Microsoft Windows, Linux, Mac OS, used to replicate the study result. ( SCIRP) ( http://www.rstudio.com/).

### Ethical considerations

This study received ethical approval from the Armauer Hansen Research Institute (AHRI) and the All-African Tuberculosis, Leprosy Treatment, Rehabilitation, and Training Centre Institutional Review Board (AHRI/ALERT IRB) with approval reference number PO/43/20 dated on 21/01/2021. Additionally, the Ministry of Education (MOE) National Ethical Review Board (NERB) of Ethiopia granted approval with reference number MOSHE/RD/04/246/84/21 dated on 10/06/2021. The study also got renewal for approval from the Ministry of Education (MOE) National Ethical Review Committee of Ethiopia with the reference number of MOSHE/RD/03/246/505/22 dated on 08/06/2022. Written informed consent was obtained from all study participants prior to their participation in the study. Within the data collection procedures, the applicant strictly maintained the participant’s confidentiality by using the study code number as identification for each participant for anonymity.

## Results

### Characteristics of the study population

A total of 300 potential participants were invited to complete the survey, 37 participants responded. Themes explored were trial sites, roles in conducted clinical trials, place of work at the time of the trial, PTA arrangements and discussions and need for PTA training.

The research team identified 242 completed clinical trials on malaria, TB, and NTDs across Sub-Saharan Africa countries. Among these, 114 trials focused on Malaria, 70 on tuberculosis, and 58 on NTDs as shown in Table 1. These clinical trials were conducted between 2008 and 2019 and were registered on clinical trials.gov or the Pan African clinical trial registry ( PACTR).

**Figure 1.**
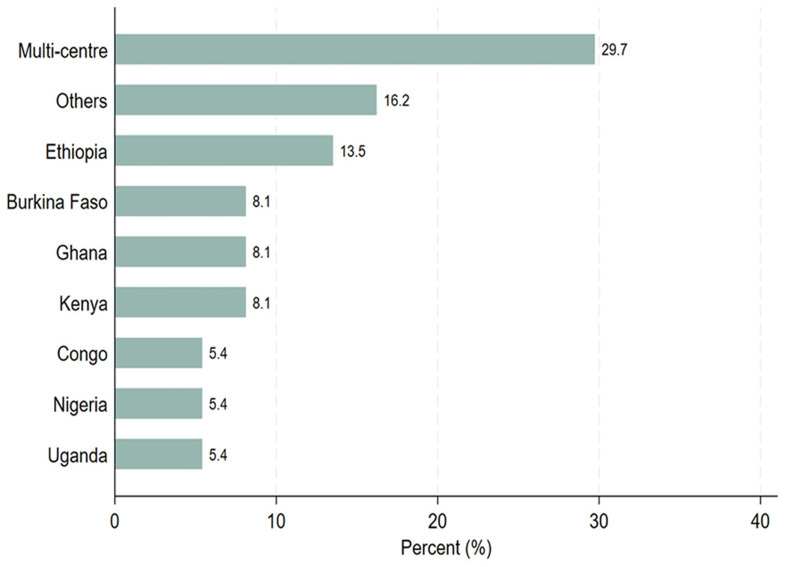
Distribution of the trial study sites, where the trial took place.

Table 2 shows the distribution of the study participants and trial sites stratified by PTA arrangement. As shown in the Table 2, thirty-seven study participants completed the survey questionnaires. Out of the 37 study participants, 21 (56.8%) were principal investigators, 4 (10.8%) were sponsors, and 12 (32.4%) were study coordinators. Most of the PIs (57.1%) and sponsors (75.0%) were involved in PTA arrangements, whereas study coordinators were evenly distributed between those who facilitated PTA arrangements and those who did not. Twenty-six out of 37 participants (70.3%) indicated that they would like to receive PTA training. Among those who would like to be trained on PTA, 38.5% had facilitated PTA arrangements. Regarding trial sites, 70% conducted single-center clinical trials, whereas 30% carried out multi-center clinical trials across sub-Saharan African countries as illustrated in Figure 1. Six of the seven known multi-center sites participated in PTA arrangements as illustrated in Table 2.

**Table 2.** Distribution of the study participants stratified by PTA arrangement, (N = 37).

	PTA arrangement		Total	*^1^P*-value
	Yes (n = 21)	No (n = 16)		
**Role of the participants: n (%)**				0.72
Principal Investigator (PI)	12 (57.1)	9 (42.9)	21	
Sponsor	3 (75.0)	1 (25.0)	4	
Study co-ordinator	6 (50.0)	6 (50.0)	12	
Need for PTA training				< 0.01
Yes	10 (38.5)	16 (61.5)	26	
No	11 (100.0)	0 (0.0)	11	
**Trial sites**				
**Single-center: n (%)**				0.94
Burkina Faso	2 (66.7)	1 (33.3)	3	
Cote d'Ivoire	0 (0.0)	1 (100.0)	1	
Democratic Republic of Congo	2 (100.0)	0 (0.0)	2	
Ethiopia	2 (40.0)	3 (60.0)	5	
Ghana	1 (33.3)	2 (66.7)	3	
Kenya	2 (66.7)	1 (3.3)	3	
Malawi	0 (0.0)	1 (100.0)	1	
Mozambique	1 (100.0)	0 (0.0)	1	
Nigeria	1 (50.0)	1 (50.0)	2	
Senegal	1 (100.0)	0 (0.0)	1	
South Africa	1 (100.0)	0 (0.0)	1	
Tanzania	0 (0.0)	1 (100.0)	1	
Uganda	1 (50.0)	1 (50.0)	2	
**Multi-center: n (%)**				
Benin, Burkina Faso, Ethiopia, Kenya, Uganda, Malawi, Tanzania, Mali	1 (100.0)	0 (0.0)	1	
Ethiopia, Kenya, Sudan, Uganda	1 (100.0)	0 (0.0)	1	
Tanzania, Equatorial Guinea	1 (100.0)	0 (0.0)	1	
South Africa, Tanzania, Mozambique, The Gambia	0 (0.0)	1 (100.0)	1	
The Gambia, Sierra Leone, Senegal	1 (100.0)	0 (0.0)	1	
Kenya, Uganda, Mozambique, Rwanda, Mali, Burkina-Faso, Gabon	1 (100.0)	0 (0.0)	1	
The Gambia, Senegal, Nigeria, and Guinea Conakry	1 (100.0)	0 (0.0)	1	
**^2^Unknown center (s)**	1 (25.0)	3 (75.0)	4	
**Trial site by size summarized: n (%)**				0.43
Multi-centered	7 (63.6)	4 (36.4)	11	
Single-centered	14 (53.9)	12 (46.1)	26	

^1^Fisher’s exact *P*-value showing the association between the role of the participants, PTA training and trial site with PTA arrangements. For example, the association between role and PTA arrangements has a P = 0.72. This means that the PIs, sponsors, and study co-ordinators were equally likely to make PTA arrangements. ^2^The names of the participating countries are not given.

### Post-trial access plans and arrangements

**Figure 2.**
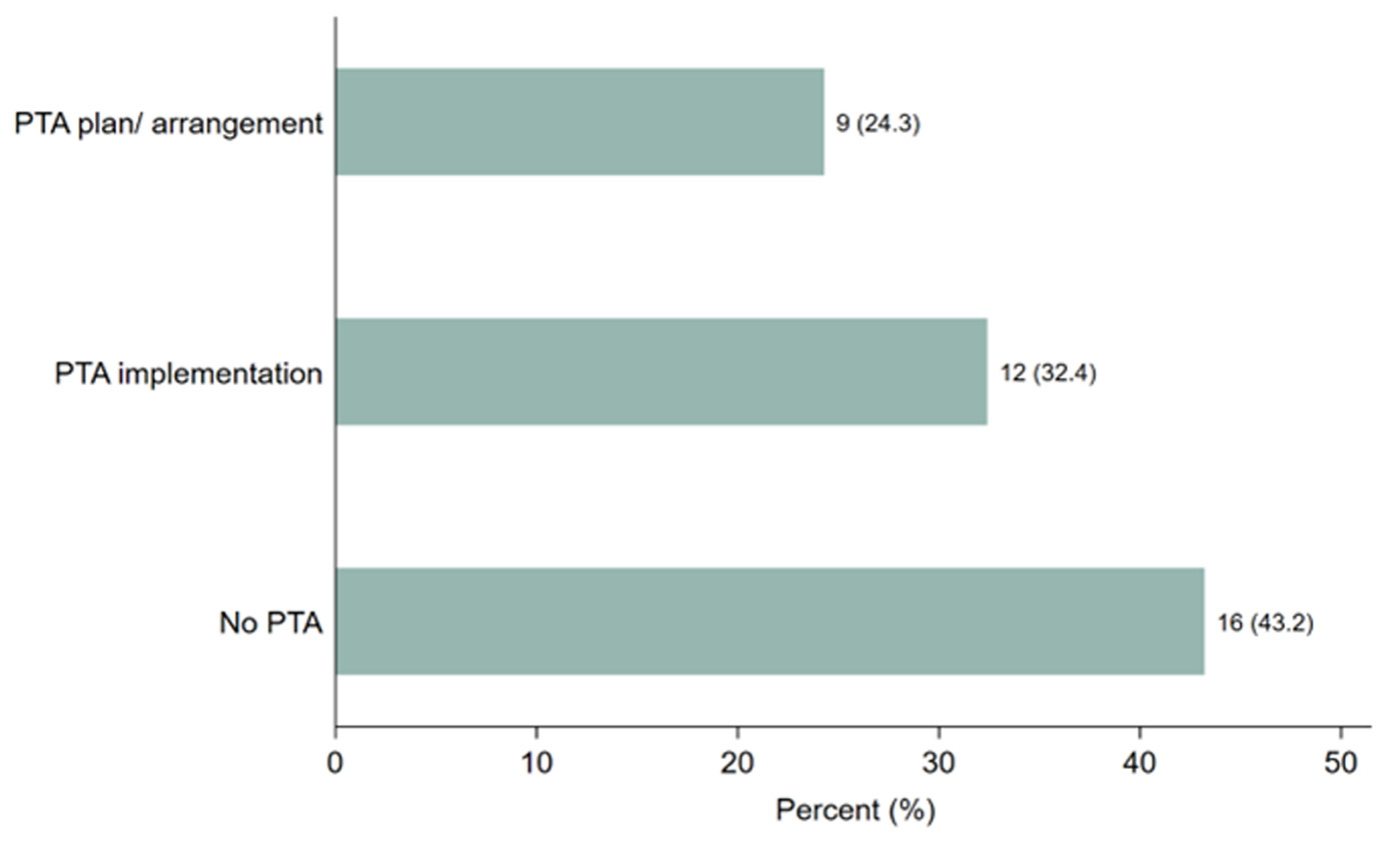
Distribution of the responses on PTA plans, discussion or arrangements made.

Respondents were asked whether they provided PTA plans, conducted discussions, or made arrangements in their clinical trials and, if yes, in what form. As shown in Figure 2, 21 (57%) study participants stated that they provided PTA plans, discussions, or arrangements. Figure 2 illustrates that 9 participants (24.3%) had developed PTA plans or arrangements, while 12 (32.4%) had implemented PTA. However, 16 participants (43%) reported no discussions or arrangements made regarding PTA in clinical trials related to malaria, tuberculosis, or neglected tropical diseases (NTDs) across Sub-Saharan African countries. These findings highlight a significant gap in PTA planning and provision, despite the steady increase in clinical trial studies conducted in the region within the last two decades.

### PTA training needs

**Figure 3.**
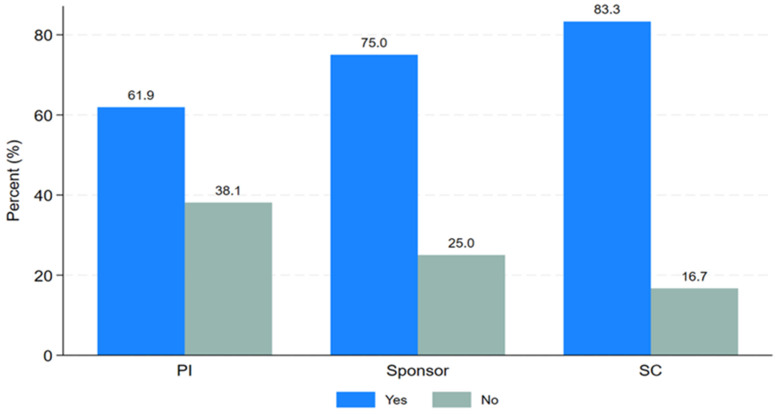
Clustered bar graph showing the desire for PTA training.

PTA arrangements involve a process initiated by the sponsor, funder, or host country after the conclusion of a clinical trial to provide access to information or knowledge about the research, or newly identified drugs, vaccines, or medical devices. PTA training, on the other hand, refers to a short course focusing on planning, types, and implementation of research findings for the community or trial participants after the clinical trial ends. Study participants were asked about their need for PTA training. Twenty-six respondents (70.3%) expressed the need for PTA training, while eleven respondents (29.7%) were not interested as shown in Table 2. Most respondents were keen to learn about PTA planning, arrangements, and implementation. As shown in " Figure 3," the majority of Principal Investigators (61.9%), sponsors (75%), and study coordinators (83.3%) indicated a desire for PTA training.

## Discussion

This study explore PTA planning, arrangements, and implementation practice in TB, Malaria and NTDs clinical trial studies conducted in SSA. The findings are key as they provide a glimpse of PTA, an underexplored concept in clinical trial research within SSA. Although more than half of the surveyed participants stated that clinical trials had PTA plans, discussions or arrangements (57.14%), only 32.4% reported implementing PTA.

These numbers indicate low PTA. However, they are slightly higher than the earlier findings of ( Hellmann *et al.*, 2022), which reported no PTA in LMIC and ( Jimenez *et al.*, 2019) which found that while the majority of clinical trial sponsors declared PTA, a closer examination of their responses revealed that no PTA was provided in the form of access to the medicinal product for research participants, the community, or the host country.

Institutional Review Board (IRB) members should request PTA plans during the review process of trial documents. The lack of a pan-African (or international) platform to follow up on whether researchers implement their PTA plans once the research is completed makes oversight difficult. In the case of at least some, if not most, of the Sub-Saharan countries, the absence of regulation and follow-up on the implementation process of PTA presents a significant challenge to its feasibility. The challenges related to PTA stem from limited knowledge, mutual agreement, and commitment from those responsible for ensuring the rights, safety, and well-being of study participants and the community.

Addressing these challenges can be achieved through PTA training for researchers, sponsors, and IRB members to update their perspectives on PTA and to consider it in the protocol development and review process. Active participation of research stakeholders in the planning, process, and implementation of PTA can maximize its feasibility in the region and help ensure the rights and benefits of research participants and their communities or countries.

## Strengths and limitations of the study

This is the first study in SSA to explore PTA arrangements and their implementation. The study provides a glimpse into PTA, an under-researched topic in clinical trial research. The limited access to clinical trial vaccines during COVID-19 for LMICs that participated in the research necessitates capacity building on PTA to ensure they benefit from research. This study indicates a gap in clinical research where nearly half of the studies do not provide PTA and a dire need for PTA training. Despite the low response rate from selected study participants, the study contributes to filling a knowledge gap on PTA overview with SSA.

## Conclusion and recommendations

PTA training should be offered to research stakeholders to address knowledge gaps in planning, discussions, and arrangements for PTA. National research guidelines should be updated to integrate PTA plans, ensuring fair access to medicinal products for study participants and communities in need. Furthermore, the principles of distributive justice and beneficence underscore the importance of mutual agreements among researchers, funders, sponsors, and host countries to guarantee PTA provision. **This highlights the need for** a collaborative framework where various stakeholders including governments, pharmaceutical companies, and global health organizations contribute to sustainable access solutions. A balanced approach is crucial to fostering innovation while ensuring that trial participants and their communities benefit from research outcomes.

## Data Availability

The data for this article consists of bibliographic references, which are included in the References section. Zenodo: Post-trial access practice in Malaria, Tuberculosis, and NTDs Clinical Trial studies in Sub-Saharan African countries, quantitative study. https://zenodo.org/doi/10.5281/zenodo.13752053 Figure 1. Distribution of the trial study sites, where the trial took place. Figure 2. Distribution of the responses on PTA plans, discussion or arrangements made. Figure 3. Clustered bar graph showing the desire for PTA training. Table 1. Search strategy . Table 2. Distribution of the study participants stratified by PTA arrangement, (N = 37). Information sheet for study participants. PTA data Excel format. Raw data survey 1 Raw data survey 2 Study survey questionnaires Data are available under the terms of the Creative Commons Attribution 4.0 International license (CC-BY 1.0).
