# Post-trial access practices in conducted clinical trials for Malaria, Tuberculosis, and Neglected Tropical Diseases (NTDs) across Sub-Saharan African countries:  A quantitative study

**DOI:** 10.12688/openreseurope.18175.5

**Published:** 2025-11-11

**Authors:** Yemisrach Seralegne, Cynthia Khamala Wangamati, Rosemarie de la Cruz Bernabe, Ibrahim Mdala, Martha Zewdie, Hawult Taye Adane

**Affiliations:** 1 Clinical trial unit, Armauer Hansen Research Institute, Addis Ababa, 1005, Ethiopia; 2 Centre for Medical Ethics, Institute of Health and Society, Faculty of Medicine, University of Oslo, Norway, Oslo, 0450, Norway; 3 Department of General Practice, Institute of Health and Society, University of Oslo, Oslo, Norway, Oslo, 0450, Norway

**Keywords:** post-trial access, clinical trial, TB, Malaria, NTDs

## Abstract

**Background:**

According to the Council of International Organizations and Medical Sciences (CIOMS) 2016, post-trial access (PTA) means ensuring that communities involved in research can benefit from the treatments, products, and knowledge developed during the study. Although laws and policies on PTA are still limited, the topic has recently gained attention as part of efforts to promote fair benefit sharing with low- and middle-income countries. In sub-Saharan Africa, where clinical trials have significantly increased over the past two decades, information on how PTA is planned and implemented remains scarce. This study examines how PTA was addressed in clinical trials for Tuberculosis (TB), Malaria, and Neglected Tropical Diseases (NTDs) conducted in the region between 2008 and 2019.

**Objective:**

The study aims to identify gaps in PTA planning and arrangements implementation, and suggest strategies for improving access to trial interventions and knowledge post-research.

**Method:**

A quantitative, cross-sectional study was conducted, using a self- administered online questionnaire to assess the PTA planning and implementation practices of Principal Investigators (PIs), co-PIs, trial coordinators, and sponsors involved in clinical trials in malaria, tuberculosis and NTDs across sub-Saharan African countries. Of the 300 invited potential participants, 37 provided complete responses.

**Findings:**

A large proportion (43%) of the study respondents did not provide PTA plans for TB, Malaria, and NTDs in clinical trials. The findings highlight an overall lack of formalized PTA policies and commitments in clinical trials for TB, Malaria, and NTDs in sub-Saharan Africa. Most of the study participants (70.3%) expressed the need for PTA training.

**Conclusion:**

Although the study offers valuable insights into PTA planning and practices, its generalizability may be limited by factors such as geographical and disease focus, reliance on self-reported data, and stakeholder representation. Despite these limitations, the study underscores an urgent need for structured PTA policy training programs, stakeholder collaboration, and effective training. Its findings can serve as a foundation for further research and policy development to enhance PTA in low-and middle-income countries (LMICs).

## Introduction

### Background

Clinical trials are research studies performed in people aimed at evaluating a medical, surgical, or behavioral intervention. They are the primary way that researchers find out if a new treatment, like a new drug or diet or medical device (for example, a pacemaker) is safe and effective in people ( Vulcano, 2017). A clinical trial is often used to learn if a new treatment is more effective and/or has less harmful side effects than the standard treatment ( NIH, 2023).

Post Trial Access (PTA) refers to the ethical imperative that requires the sponsor, researchers, and relevant public health authority, "to make available as soon as possible any intervention or product developed, and knowledge generated, for the population or community in which the research is carried out" ( CIOMS, 2016).

PTA has various aspects. First, it refers to providing knowledge and intervention, if any. Second, it refers to the potential beneficiaries, which could be research participants and/or the intended patient group in the community and/or country. Third, PTA provisions for research participants must be free of charge. PTA for the community and/or host country usually comes in the form of availability and accessibility of the medicinal product within a reasonable time. Access to an investigational intervention is justified by the principle of beneficence, “which requires researchers and sponsors to safeguard the health of participants when it is in their power to do so” ( CIOMS, 2016). It is also supported by the principle of distributive justice: participants, and by extension, the community or host country, assist researchers in generating valuable data, and, in return, researchers should ensure that participants receive the needed care to safeguard their health. In advance of a clinical trial, post-trial provisions must be arranged by sponsors and researchers to be provided by themselves, healthcare systems, or governments for all participants who still need an intervention identified as beneficial and reasonably safe in the trial. Exceptions to this requirement must be approved by a research ethics committee. Specific information about post-trial provisions must be disclosed to participants as part of informed consent ( DoH, 2024).

In line with the above principles, this manuscript will explore PTA planning, type, and implementation on TB, Malaria, and NTDs clinical trials across Sub-Saharan African countries. For the purposes of this study, we define PTA arrangements or plans as preliminary discussions, commitments, and strategies developed before or during the trial to ensure post-trial access. It includes, but not limited to: (i) financial commitments for PTA, (ii) data and knowledge sharing plans, (iii) stakeholder and community engagement activities, including meetings with government agencies, regulators, or local organizations to discuss PTA expectations, (iv) informal and formal PTA discussions and commitments among sponsors, investigators, and health authorities, (v) addressing ethical review requirements related to PTA, (vi) collaboration between academic institutions and funding agencies to discuss PTA feasibility, (vii) negotiation of intellectual property (IP) rights or licensing agreements, where applicable, and (viii) inclusion of PTA considerations in trial protocols or informed consent documents.

We define PTA implementation as concrete actions after trial completion to ensure access to the tested interventions and/or knowledge. Examples include but are not limited to: (i) preferential pricing strategies for products in trial or host countries, (ii) conducting dissemination meetings with stakeholders, (iii) publishing results in open-access journals and popular literature to ensure knowledge sharing, (iv) securing regulatory approvals for the compassionate use of the intervention, (v) integration of the intervention into national health programs within a defined timeframe, (vi) technology transfer or capacity-building initiatives in the host country, (vii) distribution of educational materials, such as training guides or films, to relevant stakeholders, and (viii) post-implementation monitoring and/or evaluation to assess the uptake of impact of PTA efforts.

Although Africa is home to nearly 20% of the global population ( Worldometer, 2025; WPR, 2025) the continent hosts less than 5% of the world’s clinical trials while bearing approximately 25% of the global disease burden ( Coulidiaty, 2025; Hwenda *et al.*, 2022). This imbalance underscores the urgent need to train research stakeholders who can strengthen clinical trial conduct and advance PTA planning and practice across the region.

## Methods

### Study design

The study is a descriptive cross-sectional study. A self-administered online questionnaire was used to explore PTA planning and implementation practices among principal investigators, sponsors, and study coordinators who implemented TB, Malaria, and NTDs clinical trial studies between 2008 and 2019. The searches are conducted from November 1, 2021, to January 31, 2022, using the Clinical trials.gov and Pan African clinical trial registry. The search terms include tuberculosis, malaria, and neglected tropical diseases (NTDs), focusing on clinical trial studies conducted between 2008 to 2019 in sub-Saharan African (SSA) countries. The results are limited to trials registered in either the Clinical trials.gov or Pan African clinical trials registry. ( PACTR; trial.gov).

We identified 242 completed clinical trial studies from the registers. From the identified studies, we selected a total of 300 participants were invited to participate in the study.

From the registry, 110 participants were registered as as PI and sponsor, 122 as PIs, and 78 as trial coordinators. A total of 37 study participants agreed to participate by filling out the questionnaires. Of the 37 participants, 21 (56.8%) were principal investigators, 4 (10.8%) were sponsors, and 12 (32.4%) were study coordinators. Most participants were based in sub-Saharan African countries (56.8%).

We developed and distributed a survey questionnaire to collect information on the PTA, types, and implementation practices of PIs, Sponsors, and trial coordinators who conducted clinical trials on TB, Malaria, or NTDs across SSA based on a literature review ( Cook, 2014).

### Study setting

We sought clinical trial studies on Malaria, TB, and NTDs within SSA conducted between 2008 and 2019. We sought these studies on clinicaltrials.gov and the Pan African Clinical Trial Registry.

### Sample and sampling strategy

The respondents were principal investigators (PIs), Co-PIs, sponsors, researchers, and clinical trial coordinators who conducted clinical trial research in TB, Malaria, and NTDs between 2008 and 2019 within SSA countries. Purposive sampling was used to select the study sample. The recruitment of study participants entailed sending emails to PIs, co-PIs, sponsors, researchers, and trial coordinators who were involved in clinical trials on Malaria, TB, & NTDs between 2008 and 2019 within SSA countries. These individuals were registered on the clinicaltrials.gov or Pan African Clinical Trial Registry websites. We retrieved their contact information from the registries and emailed consent forms and questionnaires. Some of the contact information was not useful as some participants had moved on to other organizations, making it difficult to trace them and, as such, limiting the response rate. Only 37 respondents completed the questionnaire. Online surveys are known for poor response rates in low- and middle-income countries due to internet connectivity issues ( Nayak & Narayan, 2019).

The selection of 300 participants was based on the number of completed clinical trial studies identified from two clinical trial registries, totaling 242 studies, as shown in Table 1. From these studies, we compiled a list of Principal Investigators (PIs), trial coordinators, and sponsors who were eligible to provide insights into post-trial access (PTA) practices. The final sample size of 300 was determined based on the total number of unique individuals associated with these trials, ensuring a diverse and representative pool of participants across different roles in clinical trial implementation.

**Table 1.** Search strategy.

Name of the database	Date the search was conducted	Search words	Conducted year	How combined the search term	Number of records
Clinical trials.gov & Pan African clinical trial registry	November 1, 2021 – January 31, 2022	Tuberculosis	2008 – 2019	Completed clinical trial study in sub-Saharan African countries	70
Clinical trials.gov & Pan African clinical trial registry	November 1, 2021 – January 31, 2022	Malaria	2008 – 2019	Completed clinical trial study in sub-Saharan African countries	114
Clinical trials.gov & Pan African clinical trial registry	November 1, 2021 – January 31, 2022	Neglected tropical diseases (NTDs)	2008 – 2019	Completed clinical trial study in sub-Saharan African countries	58

The search strategies used in seeking clinical trial studies on tuberculosis, malaria, and NTDs studies at the clinical trial registry and pan African clinical trial registries are disease name, the time when it was conducted and completed, and the place or country where it was conducted. We also considered PTA plan/arrangement, implementation or any challenge identified to implement PTA after the completion of trial studies.

Despite the lower-than-expected response rate, the study provides valuable insights into PTA planning and implementation. In our study, we initially invited 300 eligible participants, of whom 37 completed the survey. Therefore, for all response-based analyses, we used the total number as the denominator when calculating percentages.

The search for clinical trials on TB, Malaria, and NTDs was conducted from November 1, 2021, to January 31, 2022. The keywords used for the study were TB, Malaria, NTDs, trial ID, scientific trial title, disease name, clinical trial start time, completed time, recruitment country, PI contact address, sponsor email address, and 1st author name and address.

## Data collection tool and procedures

Based on the research objective, a questionnaire with 17 questions on socio-demographics, PTA knowledge, PTA plans, discussions and arrangements, PTA implementation, stakeholder involvement, trial products, and roles was sent out. The survey questionnaire was pre-tested among the Armauer Hansen Research Institute (AHRI) researchers to assess whether the developed tool is linguistically meaningful, whether all necessary questions are asked in the right way, and whether any other challenges may have been overlooked or undetected during the tool development process. Afterwards, the questionnaire was distributed to the study participants through their email addresses. Unfortunately, the response rate was low, so we shortened the questionnaire to encourage study participation. The participant responses were stored on the AHRI server.

The shortened questionnaire contained a few questions on (i) socio-demographics, (ii) roles in the conducted clinical trials, (iii) PTA plans, types, and implementation practices, & (iv) the need for PTA training. It was combined with information sheets and consent forms and sent to 300 email addresses.

## Data management and analysis

We applied Fisher’s Exact Test to assess the relationship between principal investigators, sponsors, or study coordinators and their responses on PTA planning and implementation. We used it to calculate the exact probability of obtaining the observed data under the assumption that there is no association between the variables (null hypothesis). This test was chosen due to the relatively small sample size and the presence of cells with expected counts less than 5. One of the main goals in inferential statistics is to generalize the findings to a larger population from which the sample is drawn. To achieve this, inferential statistics such as regression analysis, requires a large sample size. However, our data was limited to 37 participants (21 PIs, 4 sponsors, and 12 study coordinators) due to the poor response rate. Therefore, we used descriptive statistics in the form of frequencies (n) and percentages (%), and bar graphs.

StataSE18 ( https://www.stata.com) software was used to perform descriptive statistics in the form of frequencies and percentages, on anonymous data to maintain participants' confidentiality. The Fisher’s exact test assessed the relationship between two categorical variables. The findings are presented in tables and in graphical form. We considered p-values < 0.05 to be statistically significant. Though we used STATA version 18 software, we shared the dataset with an Excel sheet- which can be used to perform similar descriptive analysis, or it can be exported to other freely available statistical softwares like R ( RStudio Team, 2020). The R- or RStudio software is a freely available language and environment for statistical computing and graphics that can be downloaded to most operating systems, including Microsoft Windows, Linux, and Mac OS, and used to replicate the study result. ( SCIRP) ( http://www.rstudio.com/).

### Ethical considerations

This study received ethical approval from the Armauer Hansen Research Institute (AHRI) and the All-African Tuberculosis, Leprosy Treatment, Rehabilitation, and Training Centre Institutional Review Board (AHRI/ALERT IRB) with approval reference number PO/43/20 dated on 21/01/2021. Additionally, the Ministry of Education (MOE) National Ethical Review Board (NERB) of Ethiopia granted approval with reference number MOSHE/RD/04/246/84/21 dated on 10/06/2021. The study also got renewal for approval from the Ministry of Education (MOE) National Ethical Review Committee of Ethiopia with the reference number of MOSHE/RD/03/246/505/22 dated on 08/06/2022. Written informed consent was obtained from all study participants prior to their participation in the study. Within the data collection procedures, the applicant strictly maintained the participant’s confidentiality by using the study code number as identification for each participant for anonymity.

## Results

### Characteristics of the study population

The response rate was very low. We invited 300 potential participants and only 37 responded, giving a response rate of 12.3%. Our findings showed that the need for PTA training was significantly associated with PTA arrangements, with most participants who needed PTA training not taking part in PTA arrangements. The association between the role of participants, and trial sites with PTA arrangement was not statistically significant.

The identified completed clinical trial studies on TB, Malaria, and NTDs were 242 in total, of which 70 were TB, 114 were Malaria, and 58 were NTDs. The complete response rate was 37; of these 21 were trial principal investigators (PI), 04 were sponsors, and 12 were trial coordinators, as shown in Table 1. Themes explored were trial sites, roles in conducted clinical trials, place of work at the time of the trial, PTA arrangements and discussions, and the need for PTA training. These clinical trials were conducted between 2008 and 2019 and were registered on clinical trials.gov or the Pan African clinical trial registry ( PACTR).

**Figure 1.**
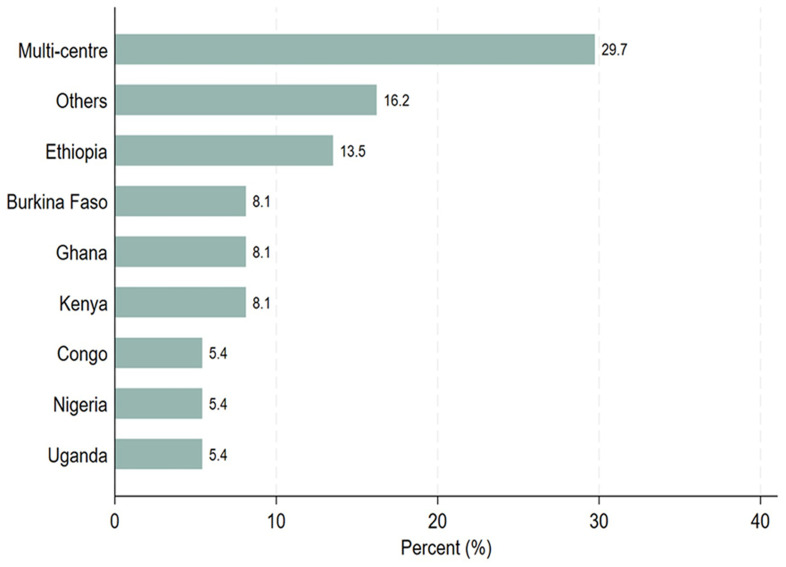
Distribution of the trial study sites, where the trial took place.

Table 2 shows the distribution of the study participants and trial sites stratified by PTA arrangement. As shown in Table 2, thirty-seven study participants completed the survey questionnaires. Out of the 37 study participants, 21 (56.8%) were principal investigators, 4 (10.8%) were sponsors, and 12 (32.4%) were study coordinators. Most of the PIs (57.1%) and sponsors (75.0%) were involved in PTA arrangements, whereas study coordinators were evenly distributed between those who facilitated PTA arrangements and those who did not. Twenty-six out of 37 participants (70.3%) indicated that they would like to receive PTA training. Among those who would like to be trained on PTA, 38.5% had facilitated PTA arrangements. Regarding trial sites, 70% conducted single-center clinical trials, whereas 30% carried out multi-center clinical trials across SSA countries as illustrated in Figure 1. Six of the seven known multi-center sites participated in PTA arrangements as illustrated in Table 2.

**Table 2.** Distribution of the study participants stratified by PTA arrangement, (N = 37).

	PTA arrangement		Total	*^1^P*-value
	Yes (n = 21)	No (n = 16)		
**Role of the participants: n (%)**				0.72
Principal Investigator (PI)	12 (57.1)	9 (42.9)	21	
Sponsor	3 (75.0)	1 (25.0)	4	
Study co-ordinator	6 (50.0)	6 (50.0)	12	
Need for PTA training				< 0.01
Yes	10 (38.5)	16 (61.5)	26	
No	11 (100.0)	0 (0.0)	11	
**Trial sites**				
**Single-center: n (%)**				0.94
Burkina Faso	2 (66.7)	1 (33.3)	3	
Cote d’Ivoire	0 (0.0)	1 (100.0)	1	
Democratic Republic of Congo	2 (100.0)	0 (0.0)	2	
Ethiopia	2 (40.0)	3 (60.0)	5	
Ghana	1 (33.3)	2 (66.7)	3	
Kenya	2 (66.7)	1 (3.3)	3	
Malawi	0 (0.0)	1 (100.0)	1	
Mozambique	1 (100.0)	0 (0.0)	1	
Nigeria	1 (50.0)	1 (50.0)	2	
Senegal	1 (100.0)	0 (0.0)	1	
South Africa	1 (100.0)	0 (0.0)	1	
Tanzania	0 (0.0)	1 (100.0)	1	
Uganda	1 (50.0)	1 (50.0)	2	
**Multi-center: n (%)**				
Benin, Burkina Faso, Ethiopia, Kenya, Uganda, Malawi, Tanzania, Mali	1 (100.0)	0 (0.0)	1	
Ethiopia, Kenya, Sudan, Uganda	1 (100.0)	0 (0.0)	1	
Tanzania, Equatorial Guinea	1 (100.0)	0 (0.0)	1	
South Africa, Tanzania, Mozambique, The Gambia	0 (0.0)	1 (100.0)	1	
The Gambia, Sierra Leone, Senegal	1 (100.0)	0 (0.0)	1	
Kenya, Uganda, Mozambique, Rwanda, Mali, Burkina-Faso, Gabon	1 (100.0)	0 (0.0)	1	
The Gambia, Senegal, Nigeria, and Guinea Conakry	1 (100.0)	0 (0.0)	1	
**^2^Unknown center (s)**	1 (25.0)	3 (75.0)	4	
**Trial site by size summarized: n (%)**				0.43
Multi-centered	7 (63.6)	4 (36.4)	11	
Single-centered	14 (53.9)	12 (46.1)	26	

^1^Fisher’s exact *P*-value showing the association between the role of the participants, PTA training and trial site with PTA arrangements. For example, the association between role and PTA arrangements has a P = 0.72. This means that the PIs, sponsors, and study co-ordinators were equally likely to make PTA arrangements. ^2^The names of the participating countries are not given.

## Post-trial access plans and arrangements

**Figure 2.**
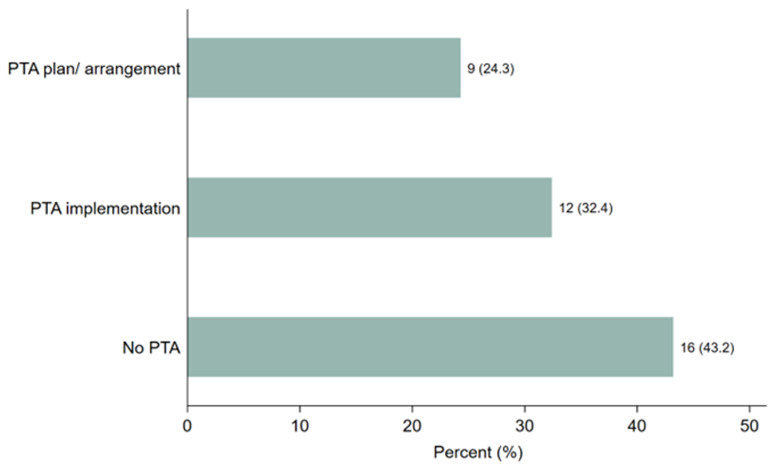
Distribution of the responses on PTA plans, discussion or arrangements made.

Respondents were asked whether they provided PTA plans, conducted discussions, or made arrangements in their clinical trials and, if yes, in what form. As shown in Figure 2, 21 (57%) study participants reported providing PTA plans, discussions, or arrangements. Figure 2 illustrates that 9 participants (24.3%) had developed PTA plans or arrangements, while 12 (32.4%) had implemented PTA. However, 16 participants (43%) reported no discussions or arrangements made regarding PTA in clinical trials related to malaria, tuberculosis, or neglected tropical diseases (NTDs) across Sub-Saharan African countries. These findings highlight a significant gap in PTA planning and provision, despite the steady increase in clinical trials conducted in the region over the last two decades.

### PTA training needs

**Figure 3.**
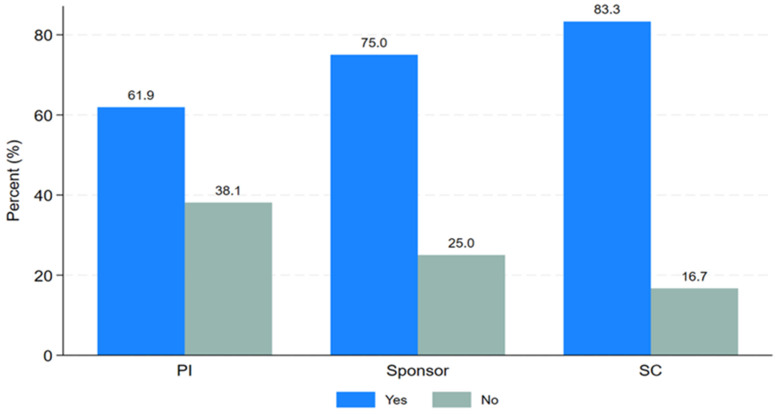
Clustered bar graph showing the desire for PTA training.

PTA arrangements involve a process initiated by the sponsor, funder, or host country after the conclusion of a clinical trial to provide access to information or knowledge about the research, or newly identified drugs, vaccines, or medical devices. PTA training, on the other hand, refers to a short course focusing on planning, types, and implementation of research findings for the community or trial participants after the clinical trial ends. Study participants were asked about their need for PTA training. Twenty-six respondents (70.3%) expressed the need for PTA training, while eleven respondents (29.7%) were not interested, as shown in Table 2. Most respondents were keen to learn about PTA planning, arrangements, and implementation. As shown in “ Figure 3,” the majority of principal investigators (61.9%), sponsors (75%), and study coordinators (83.3%) indicated a desire for PTA training.

## Discussion

This study explores PTA planning, arrangements, and implementation practice in TB, Malaria, and NTDs clinical trial studies conducted in SSA countries from 2008 to 2019.

Though only 37 respondents completed the online survey questionnaires, partly due to limited internet connectivity ( Nayak & Narayan, 2019), changes in professional roles, email addresses, or competing research commitments might be factors that likely contributed to lower than expected participation in this study. These responses still provide valuable insights. The findings highlight the limited practice of PTA, an underexplored concept in clinical trial research within SSA, across studies conducted between 2008 and 2019. **PTA plan or arrangement** carried out within the specified timeframe was very low compared to the total number of conducted trial studies, more than half of the surveyed participants stated that clinical trials had PTA plans, discussions, or arrangements (57.14%), only 32.4% reported implementing PTA. These numbers indicate low PTA. However, they are slightly higher than the earlier findings of which reported no PTA in LMIC ( Hellmann *et al*., 2022), and another study that found the majority of clinical trial sponsors declared PTA, a closer examination of their responses revealed that no PTA was provided in the form of access to the medicinal product for research participants, the community, or the host country ( Jimenez *et al*., 2019).

**PTA implementation** is defined as concrete actions after trial completion to ensure access to the tested interventions and/or knowledge. Even though, 29% of trial studies were conducted in different SSA countries, the plan and practice for PTA were very low. This confirms that there was no strong, enforceable policy, directive, or guideline requiring the PTA plan and implementation practice after completion of the trial studies. As a result, participants are exposed to unnecessary risk and exploitation, and the PTA plan and practice become less.

While 43.2 %, response accounted for **No PTA,** ascertain the plan/ arrangement and implementation practice for PTA in the region was very less, indicating triggers access to the product of the trial was overlooked in contradiction to the internation research guidelines that state that post-trial provisions must be arranged by sponsors and researchers to be provided by themselves, healthcare systems, or governments for all participants who still need an intervention identified as beneficial and reasonably safe in the trial ( CIOMS, 2016; DoH, 2024; UNESCO, 2009). This results in an imbalance in the benefit-risk ratio of the research, also contradicting the research principle of beneficence or non-maleficence which acts as the theoretical foundation of PTA and is firmly emphasized in different international ethical guidelines. Moreover, it raises the risk of exploiting study participants in low- and middle-income countries who otherwise have limited access to healthcare ( Singh *et al*., 2019). In this case, institutional and national ethics committees, as well as the relevant drug regulation agencies, must ensure that PTA is in place.

The desire **for PTA training response** showed strong interest among 62% of principal investigators, 75% of sponsors, and 83% of study coordinators. This result demonstrates the willingness of research stakeholders to seek information on PTA to update their knowledge and understanding of the topic. Providing targeted training and strengthening advocacy for PTA can play a significant role in enhancing PTA plans and practices in SSA countries. Such efforts will contribute a lot to minimizing the identified gaps in the plan, arrangement, and implementation of PTA practice in future clinical trial activities. The findings are key as they provide a glimpse of PTA, an underexplored concept in clinical trial research within SSA. Addressing these challenges requires engaging researchers, sponsors, and IRB members in training initiatives that update their perspectives on PTA and encourage its inclusion in protocol development and review processes. Active involvement of all stakeholders in planning, execution, and monitoring of PTA will enhance its feasibility while safeguarding the rights and benefits of research participants, their communities, and their countries.

## Strengths and limitations of the study

This is the first SSA study to explore PTA arrangements and their implementation. The study provides a glimpse into PTA, an under-researched topic in clinical trial research. The limited access to clinical trial vaccines during COVID-19 for LMICs that participated in the research necessitates capacity-building in PTA to ensure they benefit from research. This study indicates a gap in clinical research: nearly half of studies do not provide PTA, and there is a dire need for PTA training. Despite the low response rate among selected study participants, the study helps fill a knowledge gap regarding the PTA overview with SSA.

## Conclusion and recommendations

The study findings reveal significant gaps in PTA planning and arrangements in clinical trials conducted in SSA, alongside strong stakeholder demand for PTA training. To address these gaps, future research should incorporate mixed-method approaches, and tailored training should be provided to strengthen stakeholders’ knowledge and practices on PTA.

Furthermore, the principles of distributive justice and beneficence underscore the importance of mutual agreements among researchers, funders, sponsors, and host countries to guarantee PTA provision. This highlights the need for a collaborative framework where various stakeholders, including governments, pharmaceutical companies, and global health organizations, contribute to sustainable access solutions. A balanced approach is crucial to fostering innovation while ensuring that trial participants and their communities benefit from research outcomes.

## Data Availability

The data for this article consists of bibliographic references, which are included in the References section. Zenodo: Post-trial access practice in Malaria, Tuberculosis, and NTDs Clinical Trial studies in Sub-Saharan African countries, quantitative study. https://zenodo.org/doi/10.5281/zenodo.13752053 Figure 1. Distribution of the trial study sites, where the trial took place. Figure 2. Distribution of the responses on PTA plans, discussion or arrangements made. Figure 3. Clustered bar graph showing the desire for PTA training. Table 1. Search strategy Table 2. Distribution of the study participants stratified by PTA arrangement, (N = 37). Information sheet for study participants. PTA data Excel format. Raw data survey 1 Raw data survey 2 Study survey questionnaires Data are available under the terms of the Creative Commons Attribution 4.0 International license (CC-BY 1.0).
