# Supplementary material for: Post-trial access practices in conducted clinical trials for Malaria, Tuberculosis, and Neglected Tropical Diseases (NTDs) across Sub-Saharan African countries:  A quantitative study
Source: Open Res Eur. 2025 Nov 11;4:212. Originally published 2024 Oct 7. [Version 5] doi: 10.12688/openreseurope.18175.5 (PMC12417983; doi:10.12688/openreseurope.18175.5)
Supplement: Peer review history

# Reviewer report: Reviewer response for version 4

**DOI:** 10.21956/openreseurope.22305.r58485

**Published:** 2025-09-08

**Authors:** George Ayodo

**Affiliations:** 1 Jaramogi Oginga Odinga University of Science and Technology, Bondo, Siaya, Kenya

This is indeed a very important study on the PTA implementation to ensure access to tested intervention and /or knowledge. The study identified gaps in PTA  planning and implementation and most of the participants expressed the need for PTA training. The findings highlight the need for the formalized PTA policies and commitments. With the type of study,  there  a need to appreciate the fact that the participants are hard to reach group. Nonetheless, the study has contributed to filling gap on PTA overview in SSA.

 Here are some minor corrections

 1.The study aims to identify gaps in PTA planning and implementation, highlight challenges, and suggest strategies for improving access to trial interventions and knowledge post-research. The highlight of challenges is missing. This can be removed out of the objectives because it is missing in the result section.

 2. On the conclusion, authors should focus on the findings and implications of the study then provide recommendation to ensure generalizability.

 3. This is the spelling of sub-Sharan Africa not Sub-Saharan Africa. There should be consistent throughout the manuscript.

 4. This statement "The findings highlight critical gaps and reinforce the need for further exploration through mixed-method approaches, such as qualitative interviews or targeted stakeholder engagement, to enhance participation in future studies"  should come under recommendation not the method section. Authors should revise accordingly.

 5.  The authors should stick to PTA plans and arrangements not plans and implementation  or PTA arrangements and their implementations as indicated in the result sections.

 6. The conclusion should focus on the findings and then recommendations  for future studies to validate the findings given the low response.

Is the study design appropriate and does the work have academic merit?

Yes

Is the work clearly and accurately presented and does it cite the current literature?

Yes

If applicable, is the statistical analysis and its interpretation appropriate?

Not applicable

Are all the source data underlying the results available to ensure full reproducibility?

Yes

Are the conclusions drawn adequately supported by the results?

Yes

Are sufficient details of methods and analysis provided to allow replication by others?

Partly

Reviewer Expertise:

I am epidemiologist working on implementation research in SSA

I confirm that I have read this submission and believe that I have an appropriate level of expertise to confirm that it is of an acceptable scientific standard.

# Response

**Published:** 2025-10-30

**Authors:** Yemisrach Seralegne

**Affiliations:** clinical trial department, Armauer Hansen Research Institute, Addis Ababa, Addis Ababa, Ethiopia

**Response to  reviewer 1**   Dear Sir/Madam,   Thank you very much for your valuable comments and suggestions which is helpful to strengthen the content of our manuscript. We address all the given comments point by point as follow.   The topic of discourse is very germane given the importance it poses within the African health and research space. Below are my comments:  

**Abstract:** Well written, however, the "Background" could be made more concise.****

**Response 1. The background section of the abstract has been revised. See on page 1. The background now reads as follows: **   According to the Council of International Organizations and Medical Sciences (CIOMS) 2016, post-trial access (PTA means ensuring that communities involved in research can benefit from the treatments, products, and knowledge developed during the study. Although laws and policies on PTA are still limited, the topic has recently gained attention as part of efforts to promote fair benefit sharing with low- and middle-income countries. In Sub-Saharan Africa, where clinical trials have significantly increased over the past two decades, information on how PTA is planned and implemented remains scarce. This study examines how PTA was addressed in clinical trials for Tuberculosis (TB), Malaria, and Neglected Tropical Diseases (NTDs) conducted in the region between 2008 and 2019.      

**Introduction**: This section is overtly lengthy and should be made much concise.  

**Response 2. Thanks, we revise and make it concise**. **See on page 4 to 5. The section now reads as follows: ** Introduction **Background** Clinical trials are research studies performed in people aimed at evaluating a medical, surgical, or behavioral intervention. They are the primary way that researchers find out if a new treatment, like a new drug or diet or medical device (for example, a pacemaker) is safe and effective in people (Vulcano, 2017). A clinical trial is often used to learn if a new treatment is more effective and/or has less harmful side effects than the standard treatment (NIH, 2023). Post Trial Access (PTA) refers to the ethical imperative that requires the sponsor, researchers, and relevant public health authority, "to make available as soon as possible any intervention or product developed, and knowledge generated, for the population or community in which the research is carried out"(CIOMS, 2016). In line with the above principles, this manuscript will explore PTA planning, type, and implementation on TB, Malaria, and NTDs clinical trials across Sub-Saharan African countries. For the purposes of this study, we define PTA arrangements or plans as preliminary discussions, commitments, and strategies developed before or during the trial to ensure post-trial access. It includes, but not limited to: (i)financial commitments for PTA, (ii) data and knowledge sharing plans, (iii) stakeholder and community engagement activities, including meetings with government agencies, regulators, or local organizations to discuss PTA expectations, (iv) informal and formal PTA discussions and commitments among sponsors, investigators, and health authorities, (v) addressing ethical review requirements related to PTA, (vi) collaboration between academic institutions and funding agencies to discuss PTA feasibility, (vii) negotiation of intellectual property (IP) rights or licensing agreements, where applicable, and (viii) inclusion of PTA considerations in trial protocols or informed consent documents. We define PTA implementation as concrete actions after trial completion to ensure access to the tested interventions and/or knowledge. Examples include but are not limited to: (i) preferential pricing strategies for products in trial or host countries, (ii) conducting dissemination meetings with stakeholders, (iii) publishing results in open-access journals and popular literature to ensure knowledge sharing, (iv) securing regulatory approvals for the compassionate use of the intervention, (v) integration of the intervention into national health programs within a defined timeframe, (vi) technology transfer or capacity-building initiatives in the host country, (vii) distribution of educational materials, such as training guides or films, to relevant stakeholders, and (viii)post-implementation monitoring and/or evaluation to assess the uptake of impact of PTA efforts.  

 In 2nd paragraph, you said and I quote, "Africa is home to 17% of the world’s population and carries 25% of the global disease burden ( Hwenda *et al.* , 2022)." Please can you provide more recent reference to this citation. This section further fails to identify the justification and objectives of the study which normally comes in the penultimate and last paragraphs respectively.  

**Response 3. Noted well with thanks, we provide the recent reference to this citation. See it on page 5. ** Although Africa is home to nearly 20% of the global population, the continent hosts less than 5% of the world’s clinical trials (Makadzange, 2025) while bearing approximately 25% of the global disease burden (Coulidiaty, 2025). This imbalance underscores the urgent need to train research stakeholders who can strengthen clinical trial conduct and advance PTA planning and practice across the region.

**Study setting**: What were the search strategies used in seeking for studies in clinical trial registries?  

**Response 4. We have now briefly clarified the search strategy.  See pages 5 and 6. This reads as follows: ** Table 1. Search strategy.   Name of the database Date the search was conducted Search words Conducted year How combined the search term Number of records Clinical trial.gov & Pan African Clinical Trial Registry November 1, 2021 – January 31, 2022 Tuberculosis 2008 – 2019 Completed clinical trial study in Sub-Saharan African countries 70 Clinical trial.gov & Pan African Clinical Trial Registry November 1, 2021 – January 31, 2022 Malaria 2008 – 2019 Completed clinical trial study in Sub-Saharan African countries 114 Clinical trial.gov & Pan African Clinical Trial Registry November 1, 2021 – January 31, 2022 Neglected tropical diseases (NTDs) 2008 – 2019 Completed clinical trial study in Sub-Saharan African countries 58  

**The discussion of the factors** contributing to limited participation is inappropriate and should be moved to the discussion section accordingly.  

**Response 4. Thanks, we moved it to the discussion part now. See on page 12. The section moved to the discussion is the following: ** Discussion This study explores PTA planning, arrangements, and implementation practice in TB, Malaria, and NTDs clinical trial studies conducted in SSA countries from 2008 to 2019. Though only 37 respondents completed the online surveys questionnaires, partly due to limited internet connectivity (Nayak & Narayan, 2019), changes in professional roles, email addresses, or competing research commitments might be factors that likely contributed to lower than expected participation in this study.  These responses still provide valuable insights. The findings highlight the limited practice of PTA, an underexplored concept in clinical trial research within SSA, across studies conducted between 2008 and 2019.     

**Data management and analysis**: Please can you kindly discuss in detail the inferential analysis done.  

**Response 5. Noted well, we have provided details on how the inferential analysis done. See on page 6. This reads as follows:** **Data management and analysis**   We applied Fisher’s Exact Test to assess the relationship between principal investigators, sponsors, or study coordinators and their responses on PTA planning and implementation. We used it to calculate the exact probability of obtaining the observed data under the assumption that there is no association between the variables (null hypothesis). This test was chosen due to the relatively small sample size and the presence of cells with expected counts less than 5. One of the main goals in inferential statistics is to generalize the findings to a larger population from which the sample is drawn. To achieve this, inferential statistics such as regression analysis, requires a large sample size. However, our data was limited to 37 participants (21 PIs, 4 sponsors and 12 study coordinators) due to the poor response rate. Therefore, we used descriptive statistics in the form of frequencies (n) and percentages (%), and bar graphs.    

**Results:** What is the response rate? You claimed to have run a Fischer's exact t test but did not report it in the results section although it is recorded in table 1. I believe it appearing in the results section will be appropriate.

**Response 6. Yes, the response rate is now reported in the results section. See on page 8.** Results Characteristics of the study population The response rate was very low. We invited 300 potential participants and only 37 responded, giving a response rate of 12.3%. We agree with the reviewer that the results of the Fisher’s exact test should appear in the results section. Our findings showed that the need for PTA training was significantly associated with PTA arrangements with most participants who needed PTA training not taking part in PTA arrangements. The association between the role of participants, and trial sites with PTA arrangement was not statistically significant.   The identified completed clinical trial studies on TB, Malaria, and NTDs were 242 in total, of which 70 were TB, 114 were Malaria, and 58 were NTDs. The complete response rate was 37 of these 21 were trial principal investigators (PI), 04 were sponsors, and 12 were trial coordinators, as shown in Table 1.  

**Discussion:** This is underdeveloped and not linked to existing literatures accordingly. Therefore, develop on the discussion in line with the findings accordingly.  

**Response 7. Thank you, based on the given comment we have revised the discussion section to be in line with the findings as recommended. See on page 11 – 12. **   Discussion This study explores PTA planning, arrangements, and implementation practice in TB, Malaria, and NTDs clinical trial studies conducted in SSA countries from 2008 to 2019. Though only 37 respondents completed the online survey questionnaires, partly due to limited internet connectivity (Nayak & Narayan, 2019), changes in professional roles, email addresses, or competing research commitments might be factors that likely contributed to lower than expected participation in this study.  These responses still valuable insights. The findings highlight the limited practice of PTA, an underexplored concept in clinical trial research within SSA, across studies conducted between 2008 and 2019. 

**PTA plan or arrangement** carried out within the specified timeframe was very low compared to the total number of conducted trial studies, more than half of the surveyed participants stated that clinical trials had PTA plans, discussions or arrangements (57.14%), only 32.4% reported implementing PTA. These numbers indicate low PTA. However, they are slightly higher than the earlier findings of which reported no PTA in LMIC (Hellmann et al., 2022), and other study that found the majority of clinical trial sponsors declared PTA, a closer examination of their responses revealed that no PTA was provided in the form of access to the medicinal product for research participants, the community, or the host country (Jimenez et al., 2019).

**PTA implementation** is defined as concrete actions after trial completion to ensure access to the tested interventions and/or knowledge. Even though 29% of trial studies were conducted in different SSA countries, the plan and practice for PTA were very low. This confirms that there was no strong and enforceable policy, directive, or guideline that required the PTA plan and implementation practice after the completion of the trial studies.  While 43.2 %, response accounted for **No PTA, **  the plan/ arrangement and implementation practice for PTA in the region was  less, indicating that  access to the product of the trial was overlooked in contradiction to the internation research guidelines that state that post-trial provisions must be arranged by sponsors and researchers to be provided by themselves, healthcare systems, or governments for all participants who still need an intervention identified as beneficial and reasonably safe in the trial (CIOMS, 2016; DoH, 2024; UNESCO, 2009) . This results in an imbalance in the benefit-risk ratio of the research, also contradicting the research principle of beneficence or malfeasance, which acts as the theoretical foundation of PTA and is firmly emphasized in different international ethical guidelines.  Moreover, it raises the risk of exploiting study participants in low- and middle-income countries who otherwise have limited access to healthcare (Singh et al., 2019). In this case, institutional and national ethics committees, as well as the relevant drug regulatory agencies, must ensure that a PTA is in place. The desire **for PTA training response** showed a strong interest expressed by 62% of Principal investigators, 75% of sponsors, and 83% study coordinators. This result demonstrates the willingness of research stakeholders to seek information on PTA, updating their knowledge and understanding of the topic.  Providing targeted training and strengthening advocacy on PTA can play a significant role in enhancing the PTA plan and practice in the SSA countries. Such efforts will contribute a lot to minimizing the identified gaps in the plan, arrangement, and implementation of PTA practice in future clinical trial activities. The findings are key as they provide a glimpse of PTA, an underexplored concept in clinical trial research within SSA. Addressing these challenges requires engaging researchers, sponsors, and IRB members in training initiatives that update their perspectives on PTA and encourage its inclusion in protocol development and review processes. Active involvement of all stakeholders in planning, execution, and monitoring of PTA will enhance its feasibility while safeguarding the rights and benefits of research participants, their communities, and their countries.

**Response to reviewer 2**

**Dear Sir/Madam,** thank you very much for the given comments and suggestions that strengthen the content of our study manuscript. We address all the given comments here under point by point. 

 1. The **study aims to identify gaps in PTA planning and implementation**, highlight challenges, and suggest strategies for improving access to trial interventions and knowledge post-research. The highlight of challenges is missing. This can be removed out of **the objectives because it is missing** in the result section.

**Response 1**. We have **removed the** highlight challenges sentence from the objective list. **See on page 2.** **Objective** The study aims to identify gaps in PTA planning and arrangements implementation and suggest strategies for improving access to trial interventions and knowledge post-research.

 2 **. On the conclusion, authors should focus on the findings and implications of the study** then **provide recommendation to** ensure generalizability.  

**Response 2.** We have revised the **conclusion** part and provided recommendations to ensure generalizability. **See on page 12** **Conclusion and** **recommendations** The study findings show significant gaps in PTA planning and arrangements in clinical trials conducted in SSA, alongside a strong demand from stakeholders for PTA training. To address these gaps, future research should incorporate mixed-method approaches, and tailored training should be provided to strengthen stakeholders’ knowledge and practices on PTA.  

 3. This is the spelling of **sub-Sharan Africa not Sub-Saharan Africa**. There should be consistent throughout the manuscript.

**Response 3. **Revised as advised throughout the manuscript.    

 4. This statement " **The findings highlight critical gaps and reinforce the need for** further exploration through mixed-method approaches, such as qualitative interviews or targeted stakeholder engagement, to enhance participation in future studies” should come under recommendation not the method section. Authors should revise accordingly.

**Response 4. **Noted well with thanks, we have added this sentence in the recommendation section. **See on page 12.**

 5.  The authors **should stick to PTA plans and arrangements** **not** plans and implementation or PTA arrangements and their implementations as indicated in the result sections.

**Response 5.** Thanks, we used the PTA plan and implementation practice during the data collection process, and their response is also specific to their PTA plan, arrangement, and implementation practices. See page on 4 (method part), page 6 (Data collection tool and procedures), page 10 (post-trial access plans and arrangements), and their response on page 11 (figure 2). Methods Study design The study is a descriptive cross-sectional study.  A self-administered online questionnaire was used to explore PTA planning and implementation practices among principal investigators, sponsors, and study coordinators who implemented TB, Malaria, and NTDs clinical trial studies between 2008 and 2019. The searches are conducted from November 1, 2021, to January 31, 2022, using the Clinical trial.gov and Pan African clinical trial registry. **Data collection tool and procedures** Based on the research objective, a questionnaire with 17 questions on socio-demographics, PTA knowledge, PTA plans, discussions and arrangements, PTA implementation, stakeholder involvement, trial products, and roles was sent out. The survey questionnaire was pre-tested amongst the Armauer Hansen Research Institute (AHRI) researchers to check if the developed tool is linguistically meaningful, if all necessary questions are asked in the right way, or if any other challenges may have been overlooked or undetected during the process of tool development. Afterwards, the questionnaire was distributed to the study participants through their email addresses. Unfortunately, the response rate was low, so we shortened the questionnaire to encourage study participation. The participant responses were stored on the AHRI server. The shortened questionnaire contained a few questions on (i) socio-demographics, (ii) roles in the conducted clinical trials, (iii) PTA plans, types, and implementation practices, & (iv) the need for PTA training. It was combined with information sheets and consent forms and sent to 300 email addresses.

https://openreseurope-files.f1000.com/linked/261340.download.jfif

 Figure 2. Distribution of the responses on PTA plans, discussion or arrangements made.  

 6. The **conclusion should focus on the findings and then recommendations for future studies** to validate the findings given the low response.

**Response 6. **Thanks, we have revised the conclusion and recommended points to be considered for future clinical trial studies. **See page 12** **Discussion** This study explores PTA planning, arrangements, and implementation practice in TB, Malaria and NTDs clinical trial studies conducted in SSA countries from 2008 to 2019. Though only 37 respondents completed the online survey questionnaires, partly due to limited internet connectivity (Nayak & Narayan, 2019), changes in professional roles, email addresses, or competing research commitments might be factors that likely contributed to lower than expected participation in this study.  These responses still provide valuable insights. The findings highlight the limited practice of PTA, an underexplored concept in clinical trial research within SSA, across studies conducted between 2008 and 2019. **PTA plan or arrangement** carried out within the specified timeframe was very low compared to the total number of conducted trial studies, more than half of the surveyed participants stated that clinical trials had PTA plans, discussions or arrangements (57.14%), only 32.4% reported implementing PTA. These numbers indicate low PTA. However, they are slightly higher than the earlier findings of which reported no PTA in LMIC (Hellmann et al., 2022), and other study that found the majority of clinical trial sponsors declared PTA, a closer examination of their responses revealed that no PTA was provided in the form of access to the medicinal product for research participants, the community, or the host country (Jimenez et al., 2019). **PTA implementation** is defined as concrete actions after trial completion to ensure access to the tested interventions and/or knowledge. Even though 29% of trial studies were conducted in different SSA countries, the plan and practice for PTA were very low. This confirms that there was no strong and enforceable policy, directive, or guideline that required the PTA plan and implementation practice after the completion of trial studies. As a result, participants are exposed to unnecessary risk and exploitation, and the PTA plan and practice become less. While 43.2 %, response accounted for **No PTA, ** ascertain the plan/ arrangement and implementation practice for PTA in the region was very less, triggers access to the product of the trial was overlooked in contradiction to the internation research guidelines that state post-trial provisions must be arranged by sponsors and researchers to be provided by themselves, healthcare systems, or governments for all participants who still need an intervention identified as beneficial and reasonably safe in the trial (CIOMS, 2016; DoH, 2024; UNESCO, 2009) . This results in an imbalance in the benefit-risk ratio of the research, also contradicting the research principle of beneficence or malfeasance, which acts as the theoretical foundation of PTA and is firmly emphasized in different international ethical guidelines.  Moreover, it raises the risk of exploiting study participants in low- and middle-income countries who otherwise have limited access to healthcare (Singh et al., 2019). In this case, institutional and national ethics committees, as well as the relevant drug regulatory agencies, must ensure that a PTA is in place. While there is limited practical experience on PTA in clinical trials in LMICs. The desire **for PTA training response** showed a strong interest expressed by 62% of Principal investigators, 75% of sponsors and 83% study coordinators. This result demonstrates the willingness of research stakeholders to seek information on PTA, updating their knowledge and understanding of the topic.  Providing targeted training and strengthening advocacy on PTA can play a significant role in enhancing the PTA plan and practice in the SSA countries. Such efforts will contribute a lot to minimizing the identified gaps in the plan, arrangement, and implementation of PTA practice in future clinical trial activities. The findings are key as they provide a glimpse of PTA, an underexplored concept in clinical trial research within SSA. Addressing these challenges requires engaging researchers, sponsors, and IRB members in training initiatives that update their perspectives on PTA and encourage its inclusion in protocol development and review processes. Active involvement of all stakeholders in planning, execution, and monitoring of PTA will enhance its feasibility while safeguarding the rights and benefits of research participants, their communities, and their countries.   This is the first study in SSA to explore PTA arrangements and their implementation. The study provides a glimpse into PTA, an under-researched topic in clinical trial research. The limited access to clinical trial vaccines during COVID-19 for LMICs that participated in the research necessitates capacity building on PTA to ensure they benefit from the research. This study indicates a gap in clinical research where nearly half of the studies do not provide PTA and a dire need for PTA training. Despite the low response rate from selected study participants, the study contributes to filling a knowledge gap on the PTA overview with SSA.

**Conclusion and** **recommendations**: The study findings show significant gaps in PTA planning and arrangements in clinical trials conducted in SSA, alongside a strong demand from stakeholders for PTA training. To address these gaps, future research should incorporate mixed-method approaches, and tailored training should be provided to strengthen stakeholders’ knowledge and practices on PTA. Furthermore, the principles of distributive justice and beneficence underscore the importance of mutual agreements among researchers, funders, sponsors, and host countries to guarantee PTA provision. This highlights the need for a collaborative framework where various stakeholders including governments, pharmaceutical companies, and global health organizations contribute to sustainable access solutions. A balanced approach is crucial to fostering innovation while ensuring that trial participants and their communities benefit from research outcomes.

# Reviewer report: Reviewer response for version 4

**DOI:** 10.21956/openreseurope.22305.r58483

**Published:** 2025-08-20

**Authors:** Okereke Udohchukwu Promise

**Affiliations:** 1 University of Nigeria, Nsukka, Enugu, Nigeria

Thank you for the privilege to review this paper. The paper aims to identify the gaps in post-trial access (PTA) planning and implementation in order to improve access to trial interventions and knowledge post-research.

 The topic of discourse is very germane given the importance it poses within the African health and research space. Below are my comments:

 Abstract: Well written, however, the "Background" could be made more concise.

 Introduction: This section is overtly lengthy and should be made much concise. In 2nd paragraph, you said and I quote, "Africa is home to 17% of the world’s population and carries 25% of the global disease burden ( Hwenda *et al.*, 2022)." Please can you provide more recent reference to this citation. This section further fails to identify the justification and objectives of the study which normally comes in the penultimate and last paragraphs respectively.

 Study setting: What were the search strategies used in seeking for studies in clinical trial registries?

 The discussion of the factors contributing to limited participation is inappropriate and should be moved to the discussion section accordingly.

 Data management and analysis: Please can you kindly discuss in details the inferential analysis done.

 Results: What is the response rate? You claimed to have run a Fischer's exact t test but did not report it in the results section although it is recorded in table 1. I believe it appearing in the results section will be appropriate.

 Discussion: This is underdeveloped and not linked to existing literatures accordingly. Therefore develop on the discussion in line with the findings accordingly.

 General: The paper is quite important for the improvement of clinical trial access and knowledge improvement with Sub-Saharan Africa, however I believe that it will be greatly improved if these comments are addressed.

 Thank you and all the best with the work.

Is the study design appropriate and does the work have academic merit?

Yes

Is the work clearly and accurately presented and does it cite the current literature?

Partly

If applicable, is the statistical analysis and its interpretation appropriate?

Partly

Are all the source data underlying the results available to ensure full reproducibility?

Yes

Are the conclusions drawn adequately supported by the results?

Yes

Are sufficient details of methods and analysis provided to allow replication by others?

Partly

Reviewer Expertise:

Clinical research, public health, Public health dentistry, Bioethics.

I confirm that I have read this submission and believe that I have an appropriate level of expertise to confirm that it is of an acceptable scientific standard, however I have significant reservations, as outlined above.

# Reviewer report: Reviewer response for version 3

**DOI:** 10.21956/openreseurope.21875.r53559

**Published:** 2025-05-20

**Authors:** Irene Kyomuhangi

**Affiliations:** 1 Tulane School of Public Health and Tropical Medicine, New Orleans, USA; 2 Lancaster, Lancaster University, Lancaster, England, UK

Thank you to the authors for your responses to the previous questions and suggestions. I appreciate the efforts made, as many of my concerns have been addressed satisfactorily. I have a few remaining comments for your consideration below.

 Abstract:

- Please spell out ‘tuberculosis’ on first mention, followed by the abbreviation ‘TB’ thereafter.
- Consider rephrasing the findings: while 43% constitutes a large proportion, describing it as ‘nearly half’ is misleading in a quantitative analysis. It might be clearer to state it as ‘a large proportion’ or something similar.

Introduction:

- In the sentence ‘ *A clinical trial is often a clinical trial is used to learn if a new treatment is more effective*...’, please correct the grammatical error to improve clarity.

Methods:

- The keywords listed in this section do not seem to reflect the actual search terms used. Providing the exact search terms, ideally in a table format, would improve transparency. You might find this guidance helpful: https://kib.ki.se/en/search-evaluate/searching-information/presenting-search-strategy

Discussion:

- The statement ‘ *This is the first study in SSA to explore PTA planning, arrangements, and implementation*.’ is overly broad and potentially misleading. Consider removing or qualifying this claim.
- The sentence ‘ *Although more than half of the clinical trials had PTA plans, about 57.14% had implemented PTA*’ is confusing. It’s unclear which specific result the 57.14% refers to in the results section. Since the unit of measurement in this study is the number of individuals invited to participate rather than the number of clinical trials, referencing ‘57.14% of clinical trials’ can be misleading.

Is the study design appropriate and does the work have academic merit?

Partly

Is the work clearly and accurately presented and does it cite the current literature?

Partly

If applicable, is the statistical analysis and its interpretation appropriate?

Partly

Are all the source data underlying the results available to ensure full reproducibility?

Partly

Are the conclusions drawn adequately supported by the results?

Partly

Are sufficient details of methods and analysis provided to allow replication by others?

Partly

Reviewer Expertise:

Statistics and epidemiology, malaria, NTDs

I confirm that I have read this submission and believe that I have an appropriate level of expertise to confirm that it is of an acceptable scientific standard.

# Response

**Published:** 2025-06-05

**Authors:** Yemisrach Seralegne

**Affiliations:** clinical trial department, Armauer Hansen Research Institute, Addis Ababa, Addis Ababa, Ethiopia

**Response to the reviewer’s comments on V3 ** Thank you to the authors for your responses to the previous questions and suggestions. I appreciate the efforts made, as many of my concerns have been addressed satisfactorily. I have a few remaining comments for your consideration below.

 Abstract:

- Please spell out ‘ **tuberculosis’ **on first mention, followed by the abbreviation ‘TB’ thereafter. **Thank you, it is addressed now. Please refer to Page no. 1, paragraph 2**
- Consider rephrasing the findings: while 43% constitutes a large proportion, describing it as **‘nearly half’ is** misleading in a quantitative analysis. It might be clearer to state it as ‘a large proportion’ or something similar. **Thank you for your comments, the section has been revised as advised. Please refer to Page no. 2, paragraph 1**

Introduction:

- In the sentence ‘ *A clinical trial is often **a clinical trial is** used to learn if a new treatment is more effective*...’, please correct the grammatical error to improve clarity. **Noted, and correction is taken. Please refer to page no. 4 paragraph 1**

Methods:

- The keywords listed in this section **do not seem to reflect** the actual search terms used. Providing the exact search terms, ideally **in a table format**, would improve transparency. You might find this guidance helpful: https://kib.ki.se/en/search-evaluate/searching-information/presenting-search-strategy. **Thank you, we have prepared a table that shows keywords to reflect the actual search terms.** **Please see page no.** **5 paragraph 5.**

**  **Discussion:

- The statement ‘ *This is the first study in SSA to explore PTA planning, arrangements, and implementation*.’ is **overly broad and potentially misleading**. Consider removing or qualifying this claim. **Thank you, we have revised the sentence. Please see** **the discussion part, Page no. 8, paragraph 1**
- The sentence ‘ ***Although more than half of the clinical trials had PTA plans, about 57.14%*** *had implemented PTA*’ is confusing. It’s unclear which specific result the 57.14% refers to in the results section. Since the unit of measurement in this study is the number of individuals invited to participate rather than the number of clinical trials, referencing ‘57.14% of clinical trials’ can be misleading. **Thank you, we revised the sentence as advised.  Please see the discussion part, page no. 8 paragraph 1**

# Reviewer report: Reviewer response for version 2

**DOI:** 10.21956/openreseurope.21418.r51857

**Published:** 2025-03-08

**Authors:** Irene Kyomuhangi

**Affiliations:** 1 Tulane School of Public Health and Tropical Medicine, New Orleans, USA; 2 Lancaster, Lancaster University, Lancaster, England, UK

The authors have made a good effort to address the questions raised in the previous round of review. However, there are still some important details that could be improved or are currently lacking.

 The abstract lacks crucial information about the study's scope, results, and their generalizability. It does not provide details on sample sizes or any specific quantitative metrics such as numbers, percentages, or other relevant data. As a result, the reliability and generalizability of the study and its findings remain unclear. Simply stating that these details are included in the main text is insufficient; a summary of this information in the abstract is necessary.

 Sample and sampling strategy: The authors have still not indicated why a sample size of 300 was selected and invited to participate. Was there a sample size calculation? What informed the 300. Why not select 20, or 200 or 1000? It’s also a bit odd to suggest that the response rate was low because "online surveys typically have poor response rates in low- and middle-income countries due to internet connectivity problems." The target group for this study (which is made up of PIs and CoIs and donors, etc) is not likely to be impacted by such connectivity issues.

 The updates to Table 1 have improved readability but are still confusing. Please clarify that the figures in brackets represent percentages. Additionally, the denominators for these percentages are not always evident. For instance, in the ‘Need for PTA training’ section, it is unclear what the denominators for those percentages are.

 Having multiple definitions for PTA makes it hard to discern which definition aligns with which outcome. It would be helpful to clearly distinguish between these definitions and specify the corresponding results for each one. Employing more precise language when discussing these terms could improve clarity. For instance, what exactly does the ‘PTA arrangement’ refer to? Is it a dissemination meeting or workshop? What does ‘PTA implementation’ involve? And how is this different from ‘PTA arrangement’? The authors sometimes group these terms together, while at other times they use them separately in the paper, leading to confusion. Please either group all these terms together as PTA and state this clearly, or be specific about the distinction between them.

Is the study design appropriate and does the work have academic merit?

Partly

Is the work clearly and accurately presented and does it cite the current literature?

Partly

If applicable, is the statistical analysis and its interpretation appropriate?

Partly

Are all the source data underlying the results available to ensure full reproducibility?

Partly

Are the conclusions drawn adequately supported by the results?

Partly

Are sufficient details of methods and analysis provided to allow replication by others?

Partly

Reviewer Expertise:

Statistics and epidemiology, malaria, NTDs

I confirm that I have read this submission and believe that I have an appropriate level of expertise to confirm that it is of an acceptable scientific standard, however I have significant reservations, as outlined above.

# Response

**Published:** 2025-04-14

**Authors:** Yemisrach Seralegne

**Affiliations:** clinical trial department, Armauer Hansen Research Institute, Addis Ababa, Addis Ababa, Ethiopia

**Responses for the reviewer comments on the PTA V2 manuscript**

**C1.** The abstract lacks crucial information about the study's scope, results, and their generalizability. It does not provide details on sample sizes or any specific quantitative metrics such as numbers, percentages, or other relevant data. As a result, the reliability and generalizability of the study and its findings remain unclear. Simply stating that these details are included in the main text is insufficient; a summary of this information in the abstract is necessary.  

**R1. **We have revised the abstract to include information on the study's scope, results, and generalizability. Kindly see the added highlights in the abstract section on page 1.

 C2 **. Sample and sampling strategy:** The authors have still not indicated **why a sample size of 300** was selected and invited to participate. Was there a sample size calculation? What informed the 300. Why not select 20, or 200 or 1000? It’s also a bit odd to suggest that the **response rate was low** because "online surveys typically have poor response rates in low- and middle-income countries due to internet connectivity problems." The target group for this study (which is made up of PIs and CoIs and donors, etc) is not likely to be impacted by such connectivity issues.

  R2. Thank you for the comment. The selection of 300 participants was based on the number of completed clinical trial studies identified from clinical trial registries (242 studies). From these studies, we compiled a list of relevant stakeholders Principal Investigators (PIs), trial coordinators, and sponsors who were eligible to provide insights into post-trial access (PTA) practices. The final sample size of 300 was determined based on the total number of unique individuals associated with these trials, ensuring a diverse and representative pool of participants across different roles in clinical trial implementation. Kindly, see the explanation on **page 5. **

- ** Explanation for the Low Response Rate**

Despite our repeated efforts to engage study participants through more than two reminder emails and outreach via social media platforms such as Facebook, LinkedIn, and Twitter, the response rate remained low, with only 37 out of 300 participants completing the self-administered questionnaire. Kindly see the highlighted text on pages 5 and 6 for an explanation of why we had a low response rate.    

 C3. The updates to Table 1 have improved readability but are still confusing. Please clarify that the **figures in brackets represent percentages**. Additionally, **the denominators** for these percentages are not always evident. For instance, in the ‘Need for PTA training’ section, it is unclear what the denominators for those percentages are.

 R3. We acknowledge the concern regarding the clarity of denominators in the ‘Need for PTA Training’ section. We have adjusted the table, and denominators can be calculated across rows. Kindly see the revised Table **1 on page 8.**

 C4. Having multiple definitions for PTA makes it hard to discern which definition aligns with which outcome. It would be helpful to clearly distinguish between these definitions and specify the corresponding results for each one. Employing more precise language when discussing these terms could improve clarity. For instance, what exactly does the ‘PTA arrangement’ refer to? Is it a dissemination meeting or workshop? What does ‘PTA implementation’ involve? And how is this different from ‘PTA arrangement’? The authors sometimes group these terms together, while at other times they use them separately in the paper, leading to confusion. Please either group all these terms together as PTA and state this clearly or be specific about the distinction between them.  

 R4. Thank you for the comment. We have provided definitions in the introduction section. Please see the highlighted text **on page 4.**      

# Reviewer report: Reviewer response for version 1

**DOI:** 10.21956/openreseurope.19644.r48308

**Published:** 2025-01-06

**Authors:** Richard Idro

**Affiliations:** 1 Makerere University College of Health Sciences, Kampala, Uganda

First, old or outdated information is used to inform the study. For example, the quote that only 0.025% of registered trials in clinicaltrials.gov come from Africa was a study published in 2014. Similarly, that nearly 50% of African studies are conducted in South Africa is based on a publication over 12 years ago. There could have been a lot of changes since then.

 Secondly, it was not clear how a decision was made to invite 300 participants. Was this the required sample size? If so, the study failed badly as only 37 responses (12%) were obtained. What, therefore, is the validity of these findings? They cannot surely be representative.

 Moreover, even the 37 responses were based on a shorter version of the study tool, and not the initial tested questionnaire because of the poor response.

 Figure 1, which attempts to give the distribution of the respondents' country uses percentages. This gives a wrong perception for such a small number. They should have used the actual numbers. Furthermore, on the validity of the results, what was the distribution of the respondents (responded and based in an African Institution vs responded and based in a non-African Institution) to understand these issues? This comparison is not given.

 The methods left out a critical component: the investigators did not provide a standardized definition or criteria of what PTA in this context is. This was left for the respondents to figure out. It is unlikely that the responses all meant the same thing. Thus, the results are presented in non-uniform manner.

 Potentially, the ability to provide post-trial care as such is more difficult with the PI, co-PI and study coordinators than sponsors especially if these are Pharmaceutical companies. Unfortunately, only 4 of the respondents were companies. First, what proportion out of the 300 approached were companies? Was this representative? Secondly, were the 4/37 who responded similar? Could more have provided PTA if more pharmaceutical companies provided answers?

 The results section is grossly inadequate but is understandable because of the small numbers.

 This study could have further been strengthened with a qualitative component.

 The whole concept of PTA and the responsibilities of the different actors need to be re-examined. Many studies in Africa are investigated, and funding is sought from foundations and scientific grants or fellowships. It is grossly unfair to imagine that such should put in plans for free post-trial distribution or differential pricing, which is beyond their scope. Such a scenario can even kill innovations.

Is the study design appropriate and does the work have academic merit?

Partly

Is the work clearly and accurately presented and does it cite the current literature?

Partly

If applicable, is the statistical analysis and its interpretation appropriate?

Not applicable

Are all the source data underlying the results available to ensure full reproducibility?

Partly

Are the conclusions drawn adequately supported by the results?

No

Are sufficient details of methods and analysis provided to allow replication by others?

No

Reviewer Expertise:

Paediatrics, Child Health, Paediatric Neurology, Clinical trials

I confirm that I have read this submission and believe that I have an appropriate level of expertise to state that I do not consider it to be of an acceptable scientific standard, for reasons outlined above.

# Reviewer report: Reviewer response for version 1

**DOI:** 10.21956/openreseurope.19644.r45749

**Published:** 2024-11-13

**Authors:** Irene Kyomuhangi

**Affiliations:** 1 Tulane School of Public Health and Tropical Medicine, New Orleans, USA; 2 Lancaster, Lancaster University, Lancaster, England, UK

The authors of this paper seek to evaluate the planning and implementation of post-trial access for clinical trials related to TB, Malaria, and Neglected Tropical Diseases (NTDs) in sub-Saharan Africa.  Post-trial access is an important part of project management that requires careful planning and fair execution. The authors should be commended for their work on this paper. However, improving the way they present and explain their research would make it stronger. They also need to be clearer about their methods and results. The paper should be revised to correct grammar and spelling mistakes and include proper references to back up their claims, especially in the ‘Background’ section. It’s also unclear which sub-Saharan countries are part of the clinical trials included in this study, when the searches for the clinical trials took place, and what specific terms were used in those searches. It's not clear if the findings can be applied to all sub-Saharan Africa or if they represent current information.

**Specific comments:**

1. Title: Please consider re-phrasing the title to add… ‘;a quantitative study’ at the end.

**Abstract: ** 

1. There are several acronyms in the abstract that haven't been defined. Please provide definitions for these upon their first use (e.g., CIOMS, TB, NTD, PI, IRB).
2. Introduction -> The introduction contains relatively long sentences. Please consider condensing this section to allow for more content in the rest of the abstract.
3. Objective -> Please ensure that the beginning of each sentence is capitalized (this applies to the ‘Method’ and ‘Finding’ subsections as well). Additionally, please specify which sub-Saharan countries are being referred to.
4. Method -> The current description is inadequate. Please provide detailed information about the quantitative methods employed and the sample sizes for each group. For instance, "We designed a questionnaire to gather information on X [list key themes]. A total of X[number of] respondents participated, including X[number of] PIs, X[number of] coordinators, etc."
5. Finding -> The information provided in this section is insufficient. Please elaborate on the findings, specifying which themes were the most common or significant. Were these themes consistent across all surveyed groups?

**Background: **The major comment for this section is to provide sufficient evidence/references for the claims made, particularly about the state of clinical trials in sub-Saharan countries.

1. In the third paragraph, the phrase "clinical trials in Africa are considered to be in their embryonic stages" is somewhat unclear. Does this refer to the quantity or the scale of the clinical trials being conducted in African nations? Furthermore, the citation used to support this statement is over seven years old—are there any more recent statistics available?
2. In the fourth paragraph, kindly cite the original source from which the 47.3% figure originates, along with the date it was calculated, since it is over 10 years old. Additionally, it would be helpful to clarify what is meant by a "well-developed health system." At this point, it is also unclear why the authors have specifically chosen to emphasize South Africa and Ethiopia.
3. In the fifth and sixth paragraphs, please ensure consistency in the definition of Post-trial access (PTA). In the fifth paragraph, PTA is described as "the ethical imperative," while in the sixth paragraph, it is characterized as "the provision of both knowledge and intervention" and refers to "potential beneficiaries." This inconsistency makes the definition of PTA as used by the authors somewhat ambiguous.
4. In the seventh paragraph, kindly provide references for the quotations included.
5. In the ninth paragraph, please provide references to support the assertions made, such as the claim that "there is limited practical experience on PTA in clinical trials in LMICs."

**Methods: **

1. This section would benefit from a clearer definition of the variables and the reasoning behind the authors' categorization, particularly those listed in Table 1: Regarding the 'lead institute' variable, the term 'Partners' in the 'Pharmaceutical company/Partners' category is vague—could the authors provide clarification? Additionally, does the lead institute refer to the participant's current location or their location during the clinical trial? What if the participant was affiliated with an associate institute or organization rather than the lead institute? Regarding the 'funder' variable, can the authors explain the decision to categorize funders as just 'LSHTM' and 'Others'? This could be due to the nature of the data, but greater transparency would be appreciated. Does the 'origin' variable pertain to the participant's place of origin, their current location, or the location they were associated with during the clinical trial? Please clarify. What does 'PTA arrangement' specifically entail? Does it refer to a planned PTA, an implemented PTA plan, or the manner of implementation of the PTA plan? What does 'PTA training' specifically refer to—does it mean a course or workshop?
2. In the study design section, the phrase 'Cross-sectional surveys were used...' implies multiple surveys—were there indeed several, and if so, could you provide an explanation?
3. In the sample and sampling strategy section, please revise the phrase 'namely called' and include references to the databases used (a website would suffice). Additionally, specify when the search was conducted, which keywords or combinations were employed in the searches, and clarify why an initial sample size of 300 was chosen.
4. In the data collection and procedure section, please define AHRI upon its first mention.
5. In the data management and analysis section, it’s unnecessary to state: 'Although we used STATA version 18 software, we shared the dataset with Excel...' Furthermore, what do the authors mean by 'The Fisher’s exact test was used to assess the associations between categorical variables'? What exactly do the authors mean by 'associations' in this context?

**Results**:

1. This section could benefit from the inclusion of a table showing the search results, detailing the number of trials found and their distribution among TB, Malaria, and NTDs, among other categories.
2. For Table 1, please refer to the first comment regarding the 'methods' section. Could you clarify what the p-values signify for lead institute, funder, origin, and site (i.e., what significant differences are being referenced, and how do these relate to the study’s objectives)?
3. Regarding Figure 2, is this indicating the location of the participants? Where they were based during the clinical trial? Where the trial itself took place? Additionally, how does this relate to the research question?
4. For post-trial access plans and arrangements, it may be more effective to categorize this data more succinctly if possible—such as ‘PTA plan/arrangement’, ‘PTA implementation’, and ‘no PTA’—to provide a clearer overview of engagement levels with PTA. This ties back to how the authors are defining PTA.
5. In the section on PTA training needs, the authors focus on participants' willingness to receive training rather than assessing their actual training requirements.

**Discussion: **

1. The conclusion that ‘The challenges related to PTA stem from a lack of knowledge, mutual agreement, and commitment from those responsible for ensuring the rights, safety, and well-being of study participants and the community.’ is not supported by the evidence presented in this paper. Specifically in Figure 4, more than half of respondents indicated some plan for, or implementation of PTA

Is the study design appropriate and does the work have academic merit?

Partly

Is the work clearly and accurately presented and does it cite the current literature?

Partly

If applicable, is the statistical analysis and its interpretation appropriate?

Partly

Are all the source data underlying the results available to ensure full reproducibility?

Partly

Are the conclusions drawn adequately supported by the results?

Partly

Are sufficient details of methods and analysis provided to allow replication by others?

Partly

Reviewer Expertise:

Statistics and epidemiology, malaria, NTDs

I confirm that I have read this submission and believe that I have an appropriate level of expertise to state that I do not consider it to be of an acceptable scientific standard, for reasons outlined above.

# Response

**Published:** 2025-02-24

**Authors:** Yemisrach Seralegne

**Affiliations:** clinical trial department, Armauer Hansen Research Institute, Addis Ababa, Addis Ababa, Ethiopia

**(1 ^st^) Reviewer comment and point by point response** ***Specific comments:***

 C1. **Title: Please consider re-phrasing the title to add… ‘; a quantitative study’ at the end.**

 R1. Thanks for the suggestion. We have addressed this comment on page 1. Please, see the research title.

**Abstract:**

**C1.  There are several acronyms in the abstract that haven't been defined. Please provide definitions for these upon their first use (e.g., CIOMS, TB, NTD, PI, IRB).**

 R1. Thank you for the comment. We have addressed the comment on page 1 paragraph 1line 1, paragraph 4 line 2, paragraph 5 line 2, and paragraph 7 line 1.

 C2. **Introduction ->** **The introduction contains relatively long sentences. Please consider condensing this section to allow for more content in the rest of the abstract.** R2•  Thank you for the comment. As requested, the introduction part used to have condensed sentences now. Kindly see page 2, paragraphs 2, 3, 4, 5, and 6.  

 C3. **Objective -> Please ensure that the beginning of each sentence is capitalized (this applies to the ‘Method’ and ‘Finding’ subsections as well). Additionally, please specify which sub- Saharan countries are being referred to.**

 R3•   The beginning of each sentence has been capitalized in the objective, method, and findings subsections as advised.  In this study all sub-Saharan African countries are included.

 C4. **Method ->** The **current description is inadequate. Please provide detailed information about the quantitative methods employed and the sample sizes for each group. For instance, "We designed a questionnaire to gather information on X [list key themes]. A total of X [number of] respondents part22icipated, including X [number of] PIs, X[number of] coordinators, etc."**

 R4•        Detail description about the quantitative method and sample size is provided on page 4, paragraph 1,3,4 and 5 line 94 to 97, and 101 to 111.

 C5. **Finding ->** **The information provided in this section is insufficient. Please elaborate on the findings, specifying which themes were the most common or significant. Were these themes consistent across all surveyed groups?**

 R5. The most common themes were identified and the findings were elaborated on page 5, paragraph 2, line  160 to 175.

**Background:** **The major comment for this section is to provide sufficient evidence/references for the claims made, particularly about the state of clinical trials in sub-Saharan countries.**

 R6. Sufficient and updated references have been used throughout the document.  

 C1. **In the third paragraph, the phrase "clinical trials in Africa are considered to be in their embryonic stages" is somewhat unclear. Does this refer to the quantity or the scale of the clinical trials being conducted in African nations? Furthermore, the citation used to support this statement is over seven years old—are there any more recent statistics available?**

 R1 •       We have deleted this sentence and used more recent statistics as advised. Kindly, see references on page 2, paragraph 2 to 4, and line 40 to 54.

 C2. **In the fourth paragraph, kindly cite the original source from which the 47.3% figure originates, along with the date it was calculated, since it is over 10 years old. Additionally, it would be helpful to clarify what is meant by a "well-developed health system." At this point, it is also unclear why the authors have specifically chosen to emphasize South Africa and Ethiopia.**

 R2 •       We have deleted the whole sentence. The content of the whole paragraph has been updated, and recent research cited results are used as a reference. Kindly see page 2, paragraphs 2, 3, and 4.

 C3. **In the fifth and sixth paragraphs, please ensure consistency in the definition of post-trial access (PTA). In the fifth paragraph, PTA is described as "the ethical imperative," while in the sixth paragraph, it is characterized as "the provision of both knowledge and intervention" and refers to "potential beneficiaries." This inconsistency makes the definition of PTA as used by the authors somewhat ambiguous.**

 R3•        PTA is defined consistently throughout the manuscript. on page 2 paragraph 4, and line 51 to  54.

 C4. **In the seventh paragraph, kindly provide references for the quotations included**.

 R4•        Reference for the quotations sentence is included on page3 paragraph 1 and line 64. 

 C5. **In the ninth paragraph, please provide references to support the assertions made, such as the claim that "there is limited practical experience on PTA in clinical trials in LMICs."**

 R5•        References is put to support the assertions made on page 3 paragraph 2 and line 67 -70.  

**Methods:** This section would benefit from a clearer definition of the variables and the reasoning behind the authors' categorization, particularly those listed in **Table 1:** C1. **Regarding the 'lead institute' variable, the term 'Partners' in the 'Pharmaceutical company/Partners' category is vague—could the authors provide clarification?**

 R1.  Thank you, based on the given comments we remove the term from the table 1.  Which is helpful to minimize bias and transfer a clear message and ideas to the readers on PTA based on the general objective of the study.

 C2. **Please clarify. What does 'PTA arrangement' specifically entail? Does it refer to a planned PTA, an implemented PTA plan, or the manner of implementation of the PTA plan? What does 'PTA training' specifically refer to—does it mean a course or workshop?**

 R2. 'PTA arrangement' term is well clarified in page 7, paragraph 4, line 209 to 211 and PTA training on line 212 to 213

 C3. **In the study design section, the phrase 'Cross-sectional surveys were used...' implies multiple surveys—were there indeed several, and if so, could you provide an explanation?**  

 R3. It is typing error; we used one survey and corrected.

 C4. **In the sample and sampling strategy section, please revise the phrase 'namely called' and include references to the databases used (a website would suffice). Additionally, specify when the search was conducted, which keywords or combinations were employed in the searches, and clarify why an initial sample size of 300 was chosen.**

 R4. Why 300 sample size is clarified well on page 4, paragraph 3 line 100 to 104. When the search was conducted, and key words employed in the searches is clarified now on page 4, paragraph 4.

 C5. **In the data collection and procedure section, please define AHRI upon its first mention.**

 R5•        AHRI is defined now on page 4, paragraph 5 line 3 to 4.

 C6. **In the data management and analysis section, it’s unnecessary to state: 'Although we used STATA version 18 software, we shared the dataset with Excel...' Furthermore, what do the authors mean by 'The Fisher’s exact test was used to assess the associations between categorical variables'? What exactly do the authors mean by 'associations' in this context?**

 R6•        Noted well and corrected. The fisher’s exact test ^1^Fisher’s exact *P*-value showing the association between the role of the participants, PTA training and trial site with PTA arrangements. For example, the association between role and PTA arrangements has a P = 0.72. This means that the PIs, sponsors and study coordinators were equally likely to make PTA arrangements.

**Results:**

 C1. **This section could benefit from the inclusion of a table showing the search results, detailing the number of trials found and their distribution among TB, Malaria, and NTDs, among other categories.**

 R1•        one paragraph was prepared and included to show the search result on page 6, paragraph 2 and line 172 to 175.

 C2. **For Table 1, please refer to the first comment regarding the 'methods' section. Could you clarify what the p-values signify for lead institute, funder, origin, and site (i.e., what significant differences are being referenced, and how do these relate to the study’s objectives)?**  

 R2. The P-Values have been explained under the revised Table 1. Please note that variables like the lead institute and funder have been removed because this information was sourced from the trial registry, not the survey. The term origin has been revised to trial site for better comprehension.

 C3. **Regarding Figure 2, is this indicating the location of the participants? Where were they based during the clinical trial? Where the trial itself took place? Additionally, how does this relate to the research question?**

 R3 •       We apologies for the lack of clarity. The location of the participants is the place where the trial itself took place, as we tried to identify the PTA practice across the sub-Saharan African countries. Consequently, it was important to know the locations of the trials.

 C4. **For post-trial access plans and arrangements, it may be more effective to categorize this data more succinctly if possible—such as ‘PTA plan/arrangement’, ‘PTA implementation’, and ‘no PTA’—to provide a clearer overview of engagement levels with PTA. This ties back to how the authors are defining PTA.**

 R4. Thanks for the suggestion, this has been amended as requested in figure 2.

 C5. **In the section on PTA training needs, the authors focus on participants' willingness to receive training rather than assessing their actual training requirements.** R5.  We focused on the need for PTA training not willingness. We apologize for the poor grammar.

**Discussion:**

 C1.         The conclusion that ‘The challenges related to PTA stem from a lack of knowledge, mutual agreement, and commitment from those responsible for ensuring the rights, safety, and well-being of study participants and the community.’ is not supported by the evidence presented in this paper. Specifically in Figure 4, more than half of respondents indicated some plan for, or implementation of PTA

 R1. **Thanks for the comment, we have revised our conclusion accordingly.** **(2 ^nd^) Reviewer comments and point by point responses **   C1. First, old or outdated information is used to inform the study. For example, the quote that only 0.025% of registered trials in clinicaltrials.gov come from Africa was a study published in 2014. Similarly, that nearly 50% of African studies are conducted in South Africa is based on a publication over 12 years ago. There could have been a lot of changes since then. **R1. Thanks, we accepted the comment and include updated information and reference now.**

 C2. Secondly, it was not clear how a decision was made to invite 300 participants. Was this the required sample size? If so, the study failed badly as only 37 responses (12%) were obtained. What, therefore, is the validity of these findings? They cannot surely be representative.

 Moreover, even the 37 responses were based on a shorter version of the study tool, and not the initial tested questionnaire because of the poor response.

**R2. Thanks, how a decision was made to invite 300 participants is clarified on page 4 paragraph 1. As we retrieve clinical trial studies conducted within 2008-to-2019-time frame, some of the contact information was not useful as some participants had moved on to other organizations, making it difficult to trace them and as such contributed to limiting the response rate**.

 C3. Figure 1, which attempts to give the distribution of the respondents' country uses percentages. This gives a wrong perception for such a small number. They should have used the actual numbers. Furthermore, on the validity of the results, what was the distribution of the respondents (responded and based in an African Institution vs responded and based in a non-African Institution) to understand these issues? This comparison is not given.

**R3. We revise figure 1. Based on the comments given by the reviewers and the general objective of this study.  We used the actual number to give a clear perception on the proportion of each participated country.**

 C4. The methods left out a critical component: the investigators did not provide a standardized definition or criteria of what PTA in this context is. This was left for the respondents to figure out. It is unlikely that the responses all meant the same thing. Thus, the results are presented in non-uniform manner.

**R4. We included a standard definition for PTA based on international research guideline references on page1, paragraph 1 and page 2, paragraph 4.**

 C5. Potentially, the ability to provide post-trial care as such is more difficult with the PI, co-PI and study coordinators than sponsors especially if these are pharmaceutical companies. Unfortunately, only 4 of the respondents were companies. First, what proportion out of the 300 approached were companies? Was this representative? Secondly, were the 4/37 who responded similar? Could more have provided PTA if more pharmaceutical companies provided answers?

**R5.** **Providing PTA to trial study participants and communities is not solely the responsibility of sponsors or pharmaceutical companies; rather, it is the responsibility of all research stakeholders. Detail description on proportion of study participants is briefly written on page 4 paragraph 1.**

 C6. The results section is grossly inadequate but is understandable because of the small numbers. This study could have further been strengthened with a qualitative component.

**R6. We have strengthened the results section of this document by incorporating details on the study population's characteristics, the PTA plan and arrangements, and the PTA training needs of study participants.**

 C7. The whole concept of PTA and the responsibilities of the different actors need to be re-examined. Many studies in Africa are investigated, and funding is sought from foundations and scientific grants or fellowships. It is grossly unfair to imagine that such should put in plans for free post-trial distribution or differential pricing, which is beyond their scope. Such a scenario can even kill innovations.

**R7. Yes, Re-examining the concept of PTA and the responsibilities of different actors is indeed important to ensure a fair and sustainable approach. While many studies in Africa rely on funding from foundations, scientific grants, or fellowships, expecting them to implement free post-trial distribution or differential pricing may not always be feasible. However, this highlights the need for a collaborative framework where multiple stakeholders—including governments, pharmaceutical companies, and global health organizations—contribute to sustainable access solutions. A balanced approach is essential to support innovation while ensuring that trial participants and** **communities benefit from the research outcomes.**
